# Tissue Engineering with Mechanically Induced Solid‐Fluid Transitions

**DOI:** 10.1002/adma.202106149

**Published:** 2021-10-23

**Authors:** Erik Mailand, Ece Özelçi, Jaemin Kim, Matthias Rüegg, Odysseas Chaliotis, Jon Märki, Nikolaos Bouklas, Mahmut Selman Sakar

**Affiliations:** ^1^ Institute of Mechanical Engineering Ecole Polytechnique Fédérale de Lausanne Lausanne 1015 Switzerland; ^2^ Sibley School of Mechanical and Aerospace Engineering Cornell University Ithaca NY 14850 USA

**Keywords:** computational mechanics, mechanobiology, microengineering, robotics, tissue engineering

## Abstract

Epithelia are contiguous sheets of cells that stabilize the shape of internal organs and support their structure by covering their surfaces. They acquire diverse morphological forms appropriate for their specific functions during embryonic development, such as the kidney tubules and the complex branching structures found in the lung. The maintenance of epithelial morphogenesis and homeostasis is controlled by their remarkable mechanics—epithelia can become elastic, plastic, and viscous by actively remodeling cell–cell junctions and modulating the distribution of local stresses. Microfabrication, finite element modelling, light‐sheet microscopy, and robotic micromanipulation are used to show that collagen gels covered with an epithelial skin serve as shape‐programmable soft matter. The process involves solid to fluid transitions induced by mechanical perturbations, generates spatially distributed surface stresses at tissue interfaces, and is amenable to both additive and subtractive manufacturing techniques. The robustness and versatility of this strategy for engineering designer tissues is demonstrated by directing the morphogenesis of a variety of molded, carved, and assembled forms from the base material. The results provide insight into the active mechanical properties of the epithelia and establish methods for engineering tissues with sustainable architectures.

## Graphical abstract

Tissues composed of a collagenous core surrounded by an epithelial sheet serve as shape‐programmable living matter. The shaping process involves solid to fluid transitions in the epithelium induced by mechanical perturbations, and the generation of spatially distributed surface stresses at the interfaces. Optochemical and microsurgical operations generate a new shape that is autonomously stabilized by the re‐epithelization process.

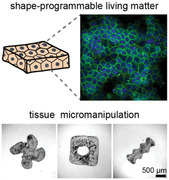

## 1 Introduction

Engineering designer tissues is instrumental for understanding embryonic development,^[^ ^1^, ^2^ ^]^ opening new avenues in regenerative medicine,^[^ ^3^, ^4^ ^]^ modelling disease biology in vitro,^[^ ^5^, ^6^, ^7^ ^]^ and building biological machines.^[^ ^8^, ^9^, ^10^ ^]^ The acquired knowledge may even inspire the construction of reconfigurable swarm robotic systems.^[^ ^11^ ^]^ However, major challenges to bringing tissue morphogenesis under engineering control arise from the multiscale dynamics of biological processes, which involve distributed intracellular forces and multicellular interactions that add up to macroscale changes in morphology.^[^ ^12^, ^13^ ^]^ Furthermore, structural and mechanical changes occur both in the self‐organizing cell collectives and the surrounding extracellular matrix (ECM) at a wide range of time scales.^[^ ^14^, ^15^ ^]^ Seminal work has shown that shaping of organs during embryonic development is coordinated by tissue mechanics,^[^ ^16^, ^17^, ^18^, ^19^, ^20^ ^]^ and engineered tissues could be programmed to recapitulate some of these morphogenic episodes.^[^ ^21^, ^22^, ^23^ ^]^

Pottery is one of the oldest human inventions. Objects with complex shapes are sculpted from clay using various additive and subtractive techniques including molding, carving, and assembling. Furthermore, the plasticity of the disordered soft material enables modelling of structures from rigid templates. Drying and firing the finished pieces inside a kiln remove the water molecules and bond the suspended particles together, essentially driving a jamming transition. Epithelial sheets have been shown to display analogous properties where the living material locally and reversibly switches from solid‐like to fluid‐like state while maintaining overall mechanical integrity.^[^ ^24^, ^25^ ^]^ The source of epithelial plasticity and fluid behavior resides in the ability to actively remodel junctions during spatiotemporally controlled proliferation, extrusion, and intercalation of constituent cells.^[^ ^26^, ^27^, ^28^, ^29^, ^30^, ^31^ ^]^ This fluidization process is a consequence of epithelial to mesenchymal and jamming transitions, which has been shown to be triggered by various factors including external mechanical loading and administration of growth factors.^[^ ^32^ ^]^

The true potential of epithelium in driving morphogenesis is revealed in the course of embryonic development with the emergence of a mesenchyme that is comprised of loosely packed migratory cells that are suspended in an extracellular matrix (ECM).^[^ ^33^, ^34^, ^35^ ^]^ A mismatch in strains between the epithelium and the underlying mesenchyme results in mechanical instabilities and tissue folding, which could be generated by the differential growth or local contraction of the tissue layers.^[^ ^16^, ^18^ ^]^ We postulated that coating the surface of a bulk fibrous gel with epithelial cells would transform the structure into a shape‐programmable living matter. The soft gel would provide a substrate on which epithelium could spread and organize, and later solidify into a thin shell that stabilizes the tissue shape. The elastic core would allow bulk mechanical manipulation as a means to drive spatially defined solid to fluid transitions in the epithelium, which would enable re‐shaping of the tissue through a sequence of plastic deformations. Local fluidization may also allow joining tissue pieces together for building more complex constructs. Finally, fluidized epithelium generates surface stresses that can deform the soft gel, therefore autonomous tissue folding could be guided along prescribed trajectories by locally perturbing the cohesion of the shell.

Here, we introduce a tissue engineering framework that harnesses mechanics of epithelia. Our method bridges the precision and versatility of traditional manufacturing techniques with the programmable and decentralized nature of the biological self‐organization. The raw composite biomaterial, consisting of collagen and epithelial cells, is cast inside microfabricated molds in a way that the cells sandwich the soft and compressible gel. Cells spread, migrate, and proliferate to cover the whole gel surface and, in the meantime, exert traction forces on the underlying matrix that are high enough to deform the bulk tissue. As the tissue compacts, increasing cell density leads to reinforcement of tight junctions and jamming where cells cannot overcome the yield stress to pass by each other and thus become immobilized. The equilibrated tissue preserves its shape for weeks. Yet, it is amenable to further shaping upon dynamic fluidization of the epithelium through mechanical and optochemical manipulations. These mechanical operations locally increase cell kinetics by creating new boundaries while transient solid to fluid transitions enable plastic forming, carving, assembly of multibody structures, and introduction of tissue folds. The experiments revealed a spectrum of 3D morphologies, demonstrating the versatility of our engineering framework.

## 2 Results

### 2.1 Fluid to Solid Transition Stabilizes the Prescribed Tissue Morphology

**Figure 1:**
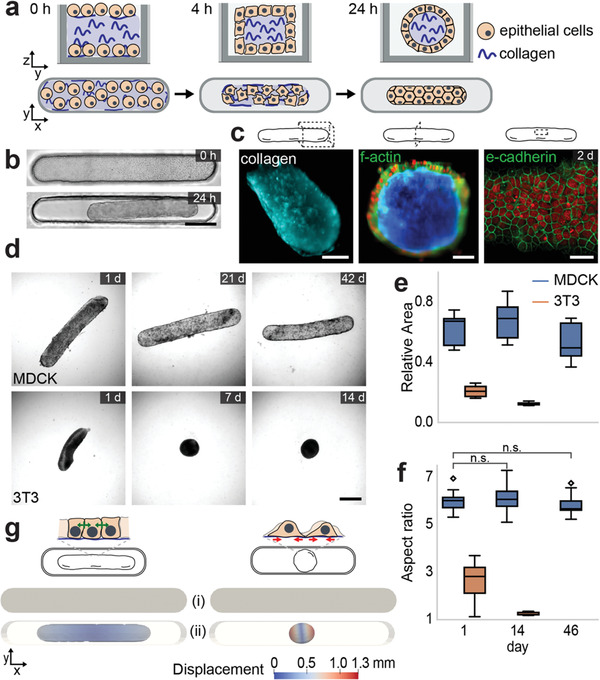
Formation of microtissues with long‐term shape stability. a) Schematic illustration of the tissue engineering process. Epithelial cells compact the polymerized collagen gel (blue) while organize the assembly of a continuous shell within 24 h. b) Representative pictures of tissues forming inside 3 × 0.4 × 0.4 mm^3^ wells. Scale bar, 500 µm. c) Microtissue architecture. (Left) 3D reconstruction of light‐sheet microscopy images showing the 3D shape of the collagen gel. Scale bar, 100 µm. (Middle) A cross‐sectional view of the tissue obtained from the reconstructed 3D image. Nuclei is shown in red, f‐actin in green, and collagen in blue. Scale bar, 50 µm. (Right) Confocal micrograph showing the epithelial monolayer at the surface of the tissue. Nuclei is shown in red, e‐cadherin is shown in green. Scale bar, 30 µm. d) Representative phase‐contrast images showing the morphological evolution of tissues over time that are formed with MDCK cells (upper panel) and 3T3 fibroblasts (lower panel). Scale bar, 500 µm. e) Boxplot showing the relative projected area over time (*N* = 3 and *n* = 12). f) Boxplot showing the aspect ratio of the tissue with respect to the body axis (*N* = 3 and *n* = 12). Tissues formed with MDCK cells are represented in blue and fibroblasts in orange. g) Finite element simulations. Schematic descriptions (upper panel) summarize the distribution of cellular forces in different tissue types. Epithelium is modelled as an elastic band which contracts with a bulk strain energy (Left). Fibroblast‐ECM interactions can be described through surface strain energy, similar to fluid‐like surface tension (Right). i) Initial state in which the contractile moduli are set to zero and ii) the equilibrium shape in which the contractile moduli are set to their calibrated values. The color bar indicates displacement magnitude in mm.

The engineering process starts with the molding of the living biomaterial, a self‐stabilizing and reconfigurable microtissue. To facilitate detailed quantitative analysis, we developed a high‐throughput device that is compatible with time‐lapse imaging using additive manufacturing and replica molding. **Figure** **1**a shows a graphical illustration of the molding process. Wells with a cylindrical geometry were filled with Madin‐Darby Canine Kidney (MDCK) sub‐type II cells^[^ ^36^ ^]^ in a suspension of reconstituted collagen type I using centrifugation and de‐wetting steps. This process ensured that cell‐laden gels were entrapped inside the wells, and the cells were dispersed close to the bottom surface of the wells. Immediately after the gelation of collagen, we seeded a second suspension of cells at a density optimized to cover the top surface. The initial seeding steps instructed the cells to populate the tissue surface within the first 24 h, a position they maintained throughout the experiments. The collagen core was compacted in 3D during this cell expansion phase (Figure 1b), where traction forces reduced the projected area of the microtissue in *x‐y* plane by 37% ± 10% (Figure 1e). The whole surface of the tissue was covered with an epithelial monolayer after 48 h in which the cells were regularly organized following the characteristic honeycomb pattern (Figure 1c and Figure S1, Supporting Information). Once reaching a steady‐state value in the course of 2–3 weeks, the size of the tissues remained the same over the next 4–5 weeks (Figure 1d,e). Notably, we did not observe any significant change in the shape of the tissues over these 7 weeks, measured by the aspect ratio with respect to the body axis in *x‐y* (Figure 1f). Our results are consistent with previous reports that have shown that MDCK cells grown on or within collagen gels in vitro maintain their epithelial character.^[^ ^37^, ^38^, ^39^ ^]^

To our surprise, the microtissues enlarged by 29% ± 12% right after the completion of the initial tissue formation phase (Figure S2, Supporting Information). We explored whether the collagenous core was under active or passive tension due to actomyosin contractility and pressure generated by the compressed gel, respectively. Enzymatic dissociation of cells from the gel caused a small enlargement of the tissue, indicating that the core was plastically deformed during compaction due to unspecific physical and electrostatic interactions among fibers. To probe the active tension on the epithelium, we used a cell‐permeable molecule to inhibit non‐muscle myosin II activity and starved cells by incubating the tissues in a buffered solution. These treatments did not lead to any detectable change in tissue shape and size (Figure S3a,b: Supporting Information). Finally, we verified that the cells stayed alive, eliminating the possibility that tissue shape remained the same due to massive cell death (Figure S3c, Supporting Information). Taken together, we conclude that the dynamic fluctuations in size that include temporary enlargement of the surface area was caused by the homeostatic activities of the epithelium that may include proliferation, active remodeling of junctions, and cell spreading.

To reveal the key role of the glassy phase transition associated with the epithelial phenotype, we performed experiments with 3T3 fibroblasts that display mesenchymal characteristics. First, we recorded time‐lapse movies of monolayers cultured on flat collagen substrates. As expected, MDCK cells drastically reduced activity upon reaching confluency, while fibroblasts continued to migrate and exchange neighbors (Figure S4 and Movie S1, Supporting Information). Next, we engineered microtissues from fibroblasts using the same molding process illustrated in Figure 1a. Fibroblasts were much more aggressive in their response and turned the tissues into almost perfect spheres within a day of culture (Figure 1d–f). We measured the surface area over time, which went down to 13% ± 1% of the initial size within 2 weeks of culture.

To further explore the mechanics behind shape stabilization, we simulated the 3D deformation of tissues by modelling the material as compressible Neo‐Hookean hyperelastic solid. We have previously shown that fibroblasts that reside on the surface of collagen gels generate surface stresses through traction forces, and we can readily capture the equilibrium tissue morphology induced by their collective mechanical action.^[^ ^40^, ^41^ ^]^ We assume that the fibroblast‐ECM interactions can be described through a surface strain energy ${\hat{\Psi }}^{a}=y\hat{J}$ that generates constant surface stresses. Based on our empirical data and literature, we hypothesized that epithelial cells form strong cell‐cell adhesion and weak cell‐ECM adhesion, building a structure reminiscent of an elastic band. The phenomenology of the problem led us to deviate from our previous approach, and model the epithelial tissue as a contractile elastic sheet surrounding the gel. The contraction of the sheet is captured by introducing a bulk strain energy Ψ^*a*^ = *ηJ*. The bulk and surface strain energies depend on the volume change ratio captured by the Jacobian of the deformation gradient *J*, and the surface area change ratio captured by the surface Jacobian $\hat{J}$. We refer the reader to Note S1 (Supporting Information) for the details of the formulation.

We developed finite element implementations of the equilibrium theory to compare the simulated shapes with the empirical data. The empirical value of Young's modulus, *E* = 200 Pa,^[^ ^42^ ^]^ corresponding to 3 mg mL^−1^ collagen was taken as an input parameter. The values of the Poisson's ratio ν, bulk contractile modulus η and surface contractile modulus γ  were fit using the experimentally measured dimensions of the tissues at the equilibrium (Table S1, Supporting Information). The simulation results recapitulated the basic observations, i.e., total collapse of tissue shape by fibroblasts and contraction of tissues while preserving aspect ratio by epithelia (Figure 1g and Figure S5, Supporting Information). The modelled epithelial tissue is 1885 µm long and the other two dimensions are 300 µm, which closely resembles the dimensions of the biological counterpart (1890 +/− 263 µm and 324 +/− 30 µm). Next, we repeated the experiments with MDCK cells at a reduced collagen concentration of 2 mg mL^−1^ (Figure S6, Supporting Information). We only changed *E* to 100 Pa^[^ ^42^ ^]^ in the model, and kept the other parameters constant. The simulation results predicted a 20% decrease in the equilibrium size, which is in close agreement with the empirical data (Table S2, Supporting Information).

### 2.2 Slip Casting of Designer Tissues

**Figure 2:**
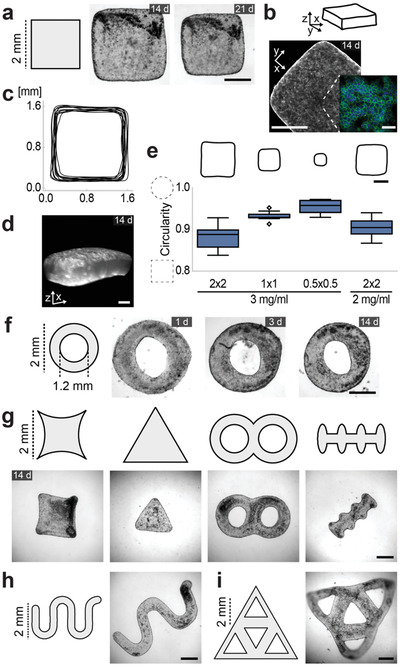
Molding of microtissues. a) Representative phase‐contrast images showing morphological evolution of tissues formed inside wells with the shape of a square prism (2 mm × 2 mm × 0.4 mm). b) Confocal micrograph of cells showing intracellular f‐actin. Scale bar, 500 µm. The inset shows f‐actin (green) and nuclei (blue) of a local region at higher magnification. Scale bar, 50 µm. c) Tissue contour lines at the *xy* plane (*n* = 12). d) 3D image of collagen gel showing the exterior profile. Scale bar, 100 µm. e) Elastocapillary effects are more emphasized in smaller tissues, and in tissues with a softer collagen core. Tissues are either molded from 3 mg mL^−1^ collagen inside wells with dimensions of 2 × 2 (2 mm × 2 mm × 0.4 mm), 1 × 1 (1 mm × 1 mm × 0.4 mm), and 0.5 × 0.5 (0.5 mm × 0.5 mm × 0.4 mm) or from 2 mg mL^−1^ collagen inside wells with dimensions of 2 × 2 (2 mm × 2 mm × 0.4 mm). Upper panel: Contour plots showing the equilibrium size. Lower panel: Boxplot showing circularity (*N* = 3 and *n* = 10). f) Representative phase‐contrast images showing morphological evolution of microtissues formed in ring‐shaped wells (depth 0.4 mm). g) Tissues formed inside wells with various geometries. h) Tissues formed inside a well with a serpentine shape. i) Tissues molded as an interconnected network of triangles. Scale bars, 500 µm.

Formation of rod‐shaped microtissues promoted the conception that the raw biomaterial may take the shape of any mold upon casting. As demonstrated by the first set of experiments, the initial gel compaction reduces the original size defined by the dimensions of the well, which must be considered in the design process. Importantly, the overall well shape is expected to be preserved unless surface stresses are non‐uniformly distributed. To systematically explore the design space, we fabricated wells with the profile of a square prism. Consistent with the previous results, the projected area of the tissues in *x‐y* plane reduced by 45% ± 5% while the aspect ratio remained at 1 ± 0.04 during the first day of culture, and the equilibrium shape remained the same over the next three weeks (**Figure** **2**a). Staining of filamentous actin revealed that the entire tissue was covered by a coherent epithelial monolayer after a week of culture (Figure 2b). The shape of the tissue contour was consistent over several samples, which presented curved corners as a manifestation of elastocapillary effects (Figure 2c). The cells were seeded only on the top and the bottom of the gel, while the sides were covered with epithelium due to cell migration. We did not observe formation of any topological features such as folds on the smooth surface of the collagen core due to the remodeling of the epithelium, and the tissue preserved the original 3D shape of the mold (Figure 2d). Notably, seeding cells only on one surface of the collagen gel caused a significant bending toward the cell‐free side of the tissue within the first day of culture, which coincided with the spreading of the epithelium toward that side (Figure S7, Supporting Information).

The scale at which surface stresses contribute to bulk morphogenesis in soft solids is defined by the elastocapillary length. This length is defined by the competition between surface forces and the bulk elastic stresses. Surface stresses (*Y*) dominate when the characteristic size of the sample is smaller than the shear elastocapillary length given by *l*_s_ = *Y*/*G* where *G* is the shear modulus.^[^ ^43^, ^44^ ^]^ Therefore, the equilibrium morphology of the microtissue, particularly at the tissue boundaries, depends on the original mold size and collagen stiffness. We fabricated square prism‐shaped molds with different sizes of base and top, while keeping the height constant. We expect the microtissue to transform into a cylinder with decreasing size, therefore circularity was chosen as the metric for evaluation. Circularity is defined as 4*πA*/*P* ^2^ where *A* is the area and *P* is the perimeter measured in *x‐y* plane. Using this formula, we can see that a circle would have a circularity measure of unity. Deviations from a circular shape means a decrease in this measure while the circularity of a square is 0.785. As expected, circularity increased with decreasing well size (Figure 2e). Along the same direction, tissues casted in identical molds but with lower collagen concentration acquired a more circular shape at the steady state.

Epithelial monolayers are capable of closing gaps by either collectively migrating over the surface in an adhesion‐dependent manner or generating traction forces parallel to the gap through the contraction of a multicellular actin purse string.^[^ ^45^, ^46^, ^47^, ^48^, ^49^ ^]^ To show the feasibility of prescribing holes into the tissues during the casting process, we engineered tissues inside a ring‐shaped mold (Figure 2f). The hole remained open over time, suggesting that the cells populated the inner walls of the hole prior to removal from the mold to form a continuous skin over the gel. To further extend the design space, we fabricated wells with a variety of convex and concave edges (Figure 2g). We observed that concave edges with low curvature are the hardest to preserve as they tend to straighten over time. One way to retain concave features is to increase aspect ratio, as demonstrated by the formation of serpentine shapes (Figure 2h). Networks formed by interconnected building blocks can realize sophisticated shapes with predetermined structural properties. In addition to the curved slender filaments, we engineered a proof‐of‐concept structure by tiling triangular unit cells within the mold (Figure 2i). The presented geometries constitute the foundation for constructing designer tissues through casting in micro fabricated molds.

### 2.3 Subtractive Manufacturing of Designer Tissues Using Microsurgery

**Figure 3:**
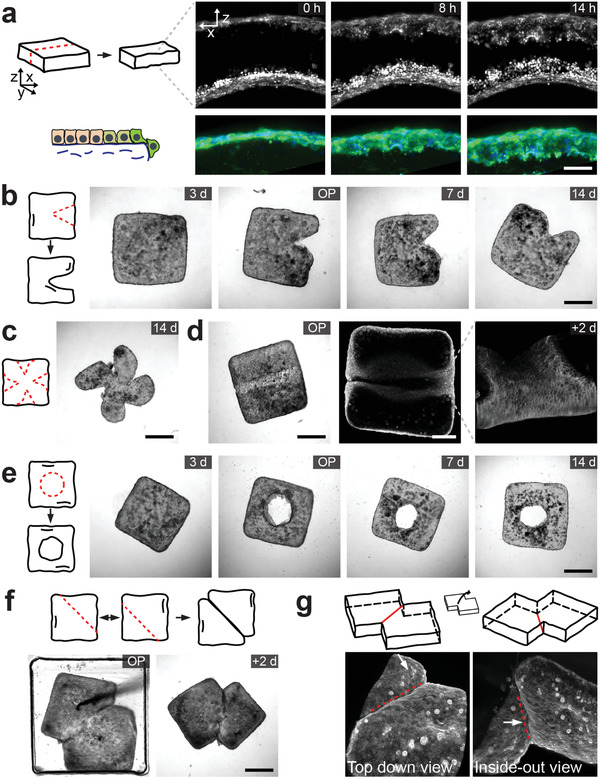
Subtractive and additive manufacturing of tissues. a) A square prism shaped tissue is cut into half using a cautery device. (Left) Schematic illustration of the cut and axes. (Right) Micrographs showing re‐epithelialization of the exposed surfaces. Closed‐up view of the re‐epithelization process is shown on the lower panel. Nuclei is shown in blue and f‐actin is shown in green. Scale bar, 100 µm. b) Shaping tissues by cutting pieces out using a microscissor or the cautery device. OP marks the images taken right after surgery. c) A representative tissue that has multiple pieces taken out surgically. d) Tissue carving using the cautery device. Phase‐contrast (left) and confocal images (right) of a carved tissue showing the valley formed on the surface by carving. Scale bars, 500 µm (left) and 200 µm (right). e) Piercing holes within tissues using a biopsy punch. f) Tissue fusion. Two cut tissue parts are brought together inside a well and maintained in physical contact at the cut surfaces. g) Light‐sheet microscopy images showing the fusion of epithelia form both sides by visualizing f‐actin. The red line indicates the side of contact. Scale bars, 500 µm (unless defined otherwise).

Experiments with insect embryos have shown that regionalized tissue fluidization is required for epithelial gap closure during gastrulation.^[^ ^50^ ^]^ Likewise, blastoderm regionally fluidizes at the onset of zebrafish development, which mediates epithelial tissue spreading over the yolk in a process called doming.^[^ ^51^ ^]^ The local fluidization of epithelial tissues promotes wound healing in Drosophila wing imaginal discs.^[^ ^29^ ^]^ We hypothesized that molded tissues could be shaped further by making mechanical manipulations to remove material, disrupting tissue boundaries in the process. The epithelium is expected to locally fluidize, and spread to cover the exposed regions of the collagen gel while preserving the prescribed shape of the bulk tissue. We used a variety of instruments to perform microsurgical operations. The first tool is a cautery device with a 13‐µm loop electrode that served as the end‐effector for carving the tissues. Dissecting a tissue into half using this instrument generated two new pieces with stable shapes. Time‐lapse imaging of labelled cells showed that cells collectively migrated to the exposed collagen gel, reconstructing the epithelial skin within a day (**Figure** **3**a and Movie S2, Supporting Information). A single full‐thickness cut performed on the tissue without modifying the bulk shape generated a defect that neither propagated nor self‐healed over time (Figure S8, Supporting Information). Next, we removed pieces of tissues by making multiple small cuts on the boundaries (Figure 3b). Several pieces could be removed from the same tissue to form more complex shapes (Figure 3c). We repeated these experiments using a more conventional tool: microscissors. We observed that the blades of the scissor compressed the tissue edges before separating the pieces from the main body, deforming the boundary to seal itself without the need for re‐epithelization. The invasive nature of this operation made it impossible to generate a straight profile at the cut edge (Figure S9, Supporting Information).

We asked whether we could carve the surface of the tissue to engrave valleys. To this end, we scratched the surface of tissues using the cautery device. Fluorescence imaging showed that cells migrated into the carved collagen surface, stabilizing the topological modification (Figure 3d). An alternative way of removing tissue pieces to form novel structures is using a punch press. We bored perforating holes into the tissues using a forged microcapillary (Figure 3e). The press had an inner tube with sharp edges so that the damage to the tissue due to mechanical loading was kept at a minimum (Figure S9, Supporting Information). In all the aforementioned manufacturing processes, a phase transition was induced by the disruption of the monolayer, essentially forcing the cells at the operated region to migrate and close the gap. With the formation of cell‐cell junctions, the new shape is frozen.

### 2.4 Assembling Multibody Tissues from Building Blocks

Self‐healing of the epithelium motivated the idea that two tissue pieces could be attached to each other. Assembly opens a design space where 3D complex morphologies are realizable.^[^ ^52^, ^53^ ^]^ The fusion process requires a prior surgical operation where we disrupt the cohesion of the epithelial shells at the interfacing boundary. This operation “activates” the surfaces that are supposed to bond together in a sense that the epithelium on each side fluidizes at the interface and flows to connect with each other. Indeed, keeping two activated pieces in physical contact with each other for 48 h was sufficient to initiate tissue fusion (Figure 3f). Gentle mechanical agitation of the assembly through pipetting or physical manipulation did not cause any detachment. Fluorescence imaging of the cells using light‐sheet microscopy verified that the cells formed a contiguous sheet around the fused tissue (Figure 3g). Repeating the assembly process without microsurgical activation did not initiate assembly, and a gentle mechanical push sufficed to detach the tissue pieces from each other (Figure S10, Supporting Information). Activating only one tissue part initiated physical bonding but with an epithelial barrier that compartmentalized the assembly at the attachment side, showing that both interfacing sides must be cut for proper fusion.

Considering the small size and delicate nature of small‐scale soft tissues, automation is instrumental for accuracy and high precision. We have recently developed a dexterous robotic manipulation system from nested piezoelectric actuators.^[^ ^40^ ^]^ The system was designed to be modular to accommodate a variety of end‐effectors, including the tools that we used to shape the tissues (Figure S11, Supporting Information). In order to make this system compatible with the presented manufacturing techniques, complex manipulation tasks that involve transport, surgery, and mechanical loading must be performed on tissues with a variety of shapes. We addressed the heterogeneity challenge by implementing a “learn from the expert” paradigm (Note S2, Supporting Information). The system is capable of recording and repeating tasks manually performed by the operator. The robot was mounted on an inverted microscope to provide visual feedback during teleoperation. We showed the feasibility of handling and carving tissues by performing pick‐and‐place tasks and making surgical cuts using actuated forceps and scissors, respectively (Figure S12 and Movie S3, Supporting Information).

### 2.5 Tissue Folding through Spatially Patterned Cell Death

**Figure 4:**
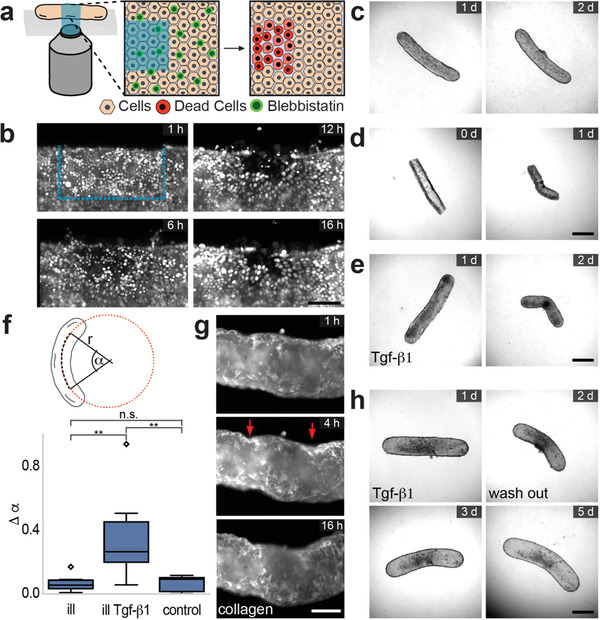
Tissue folding through programmable cell death. a) Schematic illustration of the spatiotemporally controlled cell ablation using the phototoxic activity of Blebbistatin. Illumination site is shown in blue. b) Light‐sheet microscopy images showing the nuclei of migrating cells toward the exposed area after illumination. Scale bar, 50 µm. Shape change after controlled cell death under different conditions. The cells are locally exposed to blue light c) 1 day after formation, d) 30 min after collagen polymerization, and e) 1 day after formation and subsequently treated with Tgf‐β1. Scale bars, 500 µm. f) Upper panel: Metrics to quantify tissue bending. After fitting a circle to the median line of the microtissue, the central angle α is used to compare the initial state (before illumination) and the end state (24 h later). The difference is defined as Δα = α_after_ – α_before_. Lower panel: Boxplot showing central angle of tissues exposed to light within an area of 0.3 × 0.1 mm under two different conditions and the control case where there is no illumination. The acronym ill stands for only illumination and ill Tgf‐β1 stands for illumination combined with Tgf‐β1 treatment. g) Micrographs of fluorescently labelled collagen gel showing the formation of creases at the edges of the illuminated region (red arrows). Scale bar, 100 µm. h) The shape of the tissues that are gone through illumination and Tgf‐β1 treatment are stabilized with removal of Tgf‐β1. Scale bar, 500 µm.

Local fluidization of epithelium generates a shift in force balance from cell‐cell tension to traction forces.^[^ ^54^ ^]^ We asked whether these forces could be harnessed to initiate a more drastic change in the tissue shape. Ideally, the external manipulation should activate the epithelium without damaging the underlying collagen gel. In our previous work, we introduced a technique to kill cells in a spatiotemporally resolved fashion using a photochemical conversion process.^[^ ^40^ ^]^ The technique is based on the phototoxicity of Blebbistatin, which is induced by blue light illumination. After treating tissues with Blebbistatin, we illuminated a local region at the central boundary of the tissue using a programmable digital micromirror device (**Figure** **4**a). Time‐lapse microscopy has shown that the epithelium actively moved into the exposed area over the course of 24 h (Figure 4b and Movie S4, Supporting Information) while migrating cells actively extruded the dead cells that were exposed to light (Figure S13, Supporting Information). The remodeling of the epithelium did not lead to bulk tissue deformation (Figure 4c). One important implication of this results is that if the tissues were under tension prior to the manipulation, they would bend in the direction of the non‐illuminated side due to local relaxation of stress. Notably, when we applied the same protocol during tissue formation (i.e., right after collagen polymerization), the tissues permanently bent toward the direction of the illuminated side (Figure 4d).

These observations led to the hypothesis that programmed re‐epithelization of an unjammed epithelium may be harnessed to re‐shape tissues. The literature supports this concept as it has been shown that unjammed epithelia applied relatively high traction forces on planar substrates. Furthermore, epithelial‐to‐mesenchymal transition (EMT) as well as partial EMT (pEMT) has been associated to cell unjamming in numerous biological studies.^[^ ^30^, ^55^, ^56^ ^]^ Implementing a strategy to unjam the epithelium on demand is instrumental. As a preliminary step toward this direction, we induced an EMT by treating the tissues with the transforming growth factor β‐1 (Tgf‐β1).^[^ ^32^, ^57^ ^]^ The growth factor altered tissue dynamics within 24 h, manifested by rapid tissue compaction resembling the behavior of tissues formed from 3T3 fibroblasts (Figure S14, Supporting Information). Combining optochemical killing of cells with the growth factor treatment induced bending of the tissues toward the direction of the illuminated side (Figure 4e,f). Imaging fluorescently‐labelled collagen revealed that creases formed on both sides of the illuminated area prior to illumination, indicating that cells at the wound edge locally compress the gel (Figure 4g and Movie S5, Supporting Information). Epithelium regained its jammed state upon removal of Tgf‐β1, therefore, the new shape was stabilized (Figure 4h).

## 3 Discussion

Embryonic tissues are commonly modelled as viscoelastic solids whose shape is governed by the minimization of free surface energy.^[^ ^58^, ^59^, ^60^ ^]^ Epithelial cells drive a number of morphological transitions during development by modulating their number, shape, adhesion to each other and to the underlying substrate, and tension.^[^ ^61^ ^]^ In this work, we focused on the glassy dynamics of epithelium and their capability to reversibly switch between a solid‐like and fluid‐like rheology.^[^ ^24^ ^]^ We introduced various techniques to modulate the mechanics of an epithelium that covers the surface of a compressible collagen gel, as a means to perform in vitro tissue engineering. Compared to alternative methods that act to program tension within gels,^[^ ^21^, ^23^ ^]^ our approach provides long‐term shape stability and enable on‐demand external guidance. The design space can be extended further by harnessing other collective features of epithelium, which would require tinkering with the material properties of the core gel. For example, cells could be locally induced to invaginate the gel, and guided to form buds, sacs, and networks of tubes.^[^ ^13^, ^62^ ^]^ This process may be guided by tuning ECM turnover and mechanics using engineered hydrogels.^[^ ^63^, ^64^ ^]^ We modified the local tension on the epithelium to morph the tissues by killing cells in a prescribed area. This approach could be extended to less invasive methods that involve optochemical and optogenetic modulation of proliferation and cellular contractility.^[^ ^65^, ^66^ ^]^ Another interesting direction is to externally modify the shape of the tissue without adding or removing parts. Plastic forming involves the deformation of the tissues with mechanical loading in a way that the structure preserves its new shape after the removal of the external stress. Upon stretching or bending, the epithelial monolayer would locally fluidize, remodel, and eventually re‐solidify. For this technique to work, the mechanical properties of the gel must be tuned properly.

We provided several important observations that collectively support the argument that prescribing a long‐lasting tissue morphology in our model system relies on the formation of a contiguous epithelial sheet. We hypothesize that the cells go through a jamming transition as they form a monolayer, which coincides with the development of strong cell–cell adhesions through E‐cadherin‐based junctional complexes. Previous work has shown that intercellular adhesions play an instrumental role in the stabilization of tissue shape. ^[^ ^67^ ^]^ Inhibition of E‐cadherin expression leads to an increase in traction forces exerted on the substrate.^[^ ^28^, ^68^, ^69^ ^]^ We hypothesize that our mechanical manipulations caused local unjamming of the epithelium, which temporarily perturbed the equilibrium state, making the tissue amenable to re‐shaping. In addition, this jamming transition raises the surface stresses to a level that could deform the bulk tissue, and we harnessed this potential for tissue engineering. Further experiments are needed to study the local dynamics of cell‐cell and cell‐ECM adhesion. Probing the intercellular tension and surface stresses will be instrumental to validate the theory. From an engineering perspective, the resolution of shaping could be drastically improved by controlling the spatiotemporal dynamics of cell cohesion using genetically engineered cells.

Shape‐programmable tissues are on demand for the construction of biological machines. These biomimetic devices allow testing of ideas regarding the design and operation principles of biological systems, and they may introduce novel functionality such as sensitivity to chemical signals, adaptation and self‐healing to the engineering domain. Majority of the existing prototypes are biohybrid constructs that rely on synthetic structures such as elastomers for stabilizing their shape.^[^ ^70^ ^]^ A recent work has shown that explants harvested from frog embryos preserve their shape and function over time.^[^ ^8^, ^71^ ^]^ We speculate that the epithelial skin that covers the heart muscle was responsible for this favorable outcome. Our shaping techniques have the potential to build robust machines from natural materials using native developmental programs. We can envision encapsulation of muscle cells inside the collagen core to introduce the capacity of internal actuation. Without synthetic structures, the whole machine can be edible, biodegradable, and self‐healing. Moreover, they may be programmed to reconfigure their shape upon external mechanical loading or chemical stimulation. Although a number of reports explored the formation of 3D muscle tissue as a means to build actuators,^[^ ^9^, ^72^, ^73^ ^]^ the organization of this process within an epithelium‐coated gel would require further work. We showed the feasibility of teleoperating a robotic micromanipulator to shape the tissues with minimal manual intervention. This process may be completely automated to further improve the resolution, complexity, and speed of manufacturing.

## 4 Experimental Section

### Cell Culture

MDCKII cells (ECACC, 62 107), NIH‐3T3 fibroblasts (ECACC, 93 061 524), and MDCKII H2B‐eGFP mCherry‐Actin cells (kindly provided by Prof. Daniel Müller (ETHZ)) were cultured in Dulbecco's modified Eagle's medium GlutaMAX (LifeTechnologies, Carlsbad, CA) supplemented with 10% fetal bovine serum (LifeTechnologies) and 1% penicillin‐streptomycin (LifeTechnologies). The cells were passaged every 2–3 days using phosphate‐buffered saline solution (PBS) and trypsin 0.25% EDTA (LifeTechnologies). All experiments were done with cells that were not kept longer than 20 passages. Cells were tested negative for mycoplasma. Blebbistatin (Sigma‐Aldrich) was dissolved in dimethyl sulfoxide at 10 × 10^−3^ m. Tgf‐β1 (Peprotech, 62107100‐21‐2UG) was reconstructed following manufacturers protocol and supplemented to the culture medium at 2 ng mL^−1^.

### Device Fabrication and Microtissue Model

Polydimethylsiloxane (PDMS, Sylgard 184; Dow‐Corning, Midland, MI) substrates were molded from 3D printed stamps (Formlabs Form 2). The devices were sterilized by soaking in 70% ethanol and exposing to ultraviolet light for 15 min. Before cell seeding, the devices were treated with 0.02% Pluronic‐F127 (Sigma‐Aldrich, St. Louis, MO) in water for 20 min at room temperature. 1 million cells were suspended in liquid neutralized collagen type I from rat tail (Corning BV Life Sciences) and seeded in the device. The entire assembly was centrifuged to drive cells onto the bottom of the wells. Excess solution was then removed, leaving the chambers filled with cell suspension. Following the polymerization of the microgels at 37°C for 10 min inside an incubator, a second suspension of cell (1 million cells per mL in culture medium) was added on top. After 24 h of culture, the microtissues were transferred to a PDMS‐coated flat‐bottomed 96‐well plate. A 0.1 mL drop of PDMS was added to each well to form round‐bottom chambers. The well‐plate was subsequently sterilized by ultraviolet exposure for 15 min and then treated with 0.02% Pluronic‐F127 solution for 20 min at room temperature to minimize cell adhesion.

### Tissue Micromanipulation

Glass capillaries were purchased from Science Products. Tools for tissue handling were purchased from Fine Science Tools. Microscissors (Advanced DSP Tip 27+ Straight Scissors, 727.53) and microtweezers (Advanced DSP Tip MAXGRIP Forceps 25+, 725.13P) are purchased from Alcon. The shaft of the tools is 27 and 25 Gauges, respectively. Carving of the microtissues was performed using a MC‐2010 microcautery instrument with 13‐μm wire electrodes (Protech International Inc., MC‐2010, 13‐Y1 wire tip cautery electrode). Piezoelectric actuators are assembled by SmarAct GmbH according to the design specifications. The design of the manipulator is based on an ophthalmic microsurgery platform introduced elsewhere.^[^ ^74^ ^]^ The different axes of motion were controlled by X (SmarAct SLC‐2460‐D‐L‐E), Y (SmarAct SLC‐2460‐O‐W‐D‐L‐E), Z (SmarAct SLC‐2460‐D‐L‐E), α (SmarAct SR‐4513‐D‐S), β (SmarAct SR‐2812‐S), and γ (SmarAct SR‐1908). Actuation of microscissors and microtweezers (Alcon) was driven by a stepper motor (Can‐Stack 15 000 series LC1574W‐12‐999, Haydon Kerk) controlled by an Arduino microprocessor. Robotic manipulation was performed on a fully‐motorized inverted microscope (Ti Eclipse, Nikon Instruments, Inc), and the teleoperation was performed using a 4x or 10x objective. The software package was implemented in μManager v1.4.23 and the device adapter code is hosted on c4science‐git (https://c4science.ch/source/usurgerydll.git). Adapters for common lab hardware can be found in the repository of μManager (www.micro‐manager.org/wiki/Device\%20Support).

### Cytotoxic Photoconversion

Microtissues were resuspended in culture medium containing 20 × 10^−6^ m blebbistatin (in dimethyl sulfoxide; Sigma‐ Aldrich). User‐defined regions of the tissues were exposed to 460 nm blue light (coolLED pE‐4000, Andover, UK) using a programmable digital micromirror device (Andor, Belfast, UK) through a Plan Apo /lamda 10X objective mounted on an inverted microscope. Light exposure was pulsed five times for 5 s with 30 s periods. Light intensity was adjusted between 1 and 100 µW cm^−2^ depending on the size of the selected region (0.06–0.9 mm^2^). After light exposure, the medium was exchanged with fresh culture medium.

### Wide‐Field Microscopy

Phase‐contrast, differential interference contrast (DIC) and fluorescent images were captured every hour for 20 h with an ORCA‐Flash4.0 digital CMOS camera (Hamamatsu, Japan) mounted on a motorized inverted microscope (Eclipse Ti, Nikon Instruments, Inc). The microscope is equipped with a Plan Fluor 10x objective and a live‐cell incubator for long‐term imaging (Life Imaging Services, Switzerland).

### Light‐Sheet Microscopy Imaging of Living Samples

Microtissues were engineered from MDCKII H2B‐eGFP mCherry‐Actin cells for visualizing intracellular F‐actin. Collagen type I was labelled with Alexa Fluor 647 NHS ester (ThermoFisher, A20006) by incubating the prepolymer with Alexa Fluor 647 NHS ester for 2 h at 4°C (1 µL of 1 mg mL^−1^ dye was added to 1 mL of 3.8 mg mL^−1^ collagen). Fluorescently‐labeled samples were mounted on a LS1 Live dual illumination light‐sheet microscope (Viventis Microscopy Sarl, Switzerland). The microscope was equipped with two 25X objectives (CFI75 Apochromat NA 1.1, Nikon) and three laser sources (488, 561, and 638 nm). The thickness of the Gaussian beam light sheet was adjusted to 2.2 µm. Images from all three channels were acquired at each position every 30 min. A frame of 2048 × 2048 pixel (710 × 710 µm) was selected with a sheet step size of 2–3 µm (301 planes). 3D views were constructed using a commercial software (Imaris, Bitplane). Fused microtissues were imaged using dual‐inverted Selective Plane Illumination Microscopy (Intelligent Imaging Innovations (3i), Inc) equipped with a 10X objective and a Hamamatsu ORCA Flash4.0 sCMOS camera. The acquisition was set to stage scanning mode with a 1.4 µm step size orthogonal to the focal plane and a scanning range of 1.4 mm across the sample was selected. 3D view was constructed using SlideBook (3i, Inc).

### Immunohistochemistry and Confocal Microscopy

Microtissues were fixed with 4% formaldehyde, permeabilized with 0.2% Triton X‐100, and blocked in 10% goat serum for 1 h at room temperature. Samples were subsequently incubated overnight at 4 °C with antibodies against e‐cadherin (ab11512; Abcam, Cambridge, UK) and detected with goat anti‐rat Alexa‐555 (1:100; A21434, Thermo Fisher Scientific) conjugated antibodies. F‐actin was detected with Alexa Fluor 488 Phalloidin (ThermoFisher, A12379) or SiR‐actin (Spirochrome) respectively. Nuclei were counterstained with Hoechst 33 342 (Thermo Fisher Scientific, Waltham, MA). Fluorescently labeled microtissues were imaged using an inverted confocal microscope (LSM 700; Zeiss, Oberkochen, Germany) equipped with a 20x objective.

### Collagen Assay

A previously reported protocol to attach 250 µm thick collagen gels to glass bottom petri‐dishes was adapted.^[^ ^75^ ^]^ Coverslips were treated with 2% aminopropyltriethoxysilane (APTES, Sigma, A3648) for 15 min while continuously replenishing the solution to counteract evaporation. After washing and air‐drying steps, the coverslips were incubated with 0.1% w/w glutaraldehyde (Sigma G5882, 1 µL of 50% glutaraldehyde in 500 µL H_2_O) for 15 min. After rinsing three times for 5 min with sterile water, the functionalized coverslips were attached to petri dishes with a 18 mm hole (Cell E&G) using a UV curable glue (Norland Products, USA). Liquid neutralized collagen type I from rat tail (Corning BV Life Sciences) diluted to a final concentration of 1 mg mL^−1^ was poured onto the center of the dish and sandwiched with a second coverslip. After incubation at 37 °C and 5% CO_2_ for 20 min, the second coverslip was gently detached from the collagen gel.

### Viability Assay

The Viability/Cytotoxicity Assay Kit (Biotium, Fremont, CA) was applied according to the manufacturer's protocol for visualizing live and dead cells. Briefly, microtissues were incubated in a staining solution composed of 2 × 10^−3^ m calcein acetoxymethyl as living cell stain (green) and 4 × 10^−3^ m ethidium homodimer III as dead cell stain (red) in PBS for 40 min. After incubation, tissues were washed three times with and resuspended in PBS.

### Image Processing

To quantify tissue morphology of the capsule‐shaped microtissues, time‐lapse images were first segmented using a semi‐automated processing software written in ImageJ. The software uses the E‐Snake algorithm (courtesy of Ricard Delgado‐Gonzalo, Biomedical Image Group (BIG), EPFL, Switzerland). Shape parameters were extracted with a custom script written in MATLAB (Mathworks, MA). In brief, the script skeletonizes the segmented tissue images to calculate the body axis line, measures tissue area, width, length and quantifies shape by fitting a circle to the median line to calculate the central angle. To quantify cell motion on 2D substrates, particle image velocimetry was performed using PIVlab written for Matlab.^[^ ^76^ ^]^ In order to avoid tracking discrete objects within the cells, images were treated with a fast Fourier transform bandpass filter 3–6 pixel wide. 64 × 64 pixel interrogation windows with a 50% overlap were used. Cross‐correlation was performed by using the fast Fourier transform method. During post‐processing, a velocity limit of around 25 pixel per frame was chosen and error‐prone vectors were filtered out using the standard deviation filter. The data was processed with a custom script in Matlab. The script accepted the interrogation window locations (*x,y*) of each frame and corresponding vectors (*u,v*) and calculated mean velocities at a given location or from the entire frame, respectively.

### Statistical Analysis

Boxplots have a central mark representing the median. The borders mark the first and third quartiles. Whiskers in boxplots extend to points that lie within 1.5 times the interquartile range. If the point is not within this range, it is determined to be an outlier and represented as a diamond symbol. Statistical significance was calculated with unpaired two‐sample *t*‐tests. *p*‐values greater than 0.05 were considered to be statistically not significant (n.s.). * *P* ≤ 0.05, ** *P* ≤ 0.01.

## Conflict of Interest

The authors declare no conflict of interest.

## Author Contributions

E.M. and M.S.S designed the experiments, E.M., E.O., and O.C. performed the experiments, E.M., M.R., and J.M. designed and implemented the robotic system. E.M. analyzed the data, J.K. and N.B. formulated and implemented the computational model, E.M. and M.S.S. wrote the manuscript with contributions from all authors, M.S.S supervised the research.

## Supporting information

Supporting information

Supporting Movie 1

Supporting Movie 2

Supporting Movie 3

Supporting Movie 4

Supporting Movie 5

## Data Availability

The data that support the findings of this study are available from the corresponding author upon reasonable request.
