# Supplementary material for: Tissue Engineering with Mechanically Induced Solid‐Fluid Transitions
Source: Adv Mater. 2021 Oct 23;34(2):2106149. doi: 10.1002/adma.202106149 (PMC11468955; doi:10.1002/adma.202106149)
Supplement: Supplementary file 1 — Supporting information [file ADMA-34-2106149-s003.pdf]

ADVANCED MATERIALS

Supporting Information

for *Adv. Mater.,* DOI: 10.1002/adma.202106149

Tissue Engineering with Mechanically Induced Solid-Fluid Transitions

*Erik Mailand, Ece Özelçi, Jaemin Kim, Matthias Rüegg, Odysseas Chaliotis, Jon Märki, Nikolaos Bouklas, and Mahmut Selman Sakar**

Supplementary Information

Tissue sculpting with mechanically induced solid-fluid transitions

Erik Mailand[1], Ece Özelçi[1], Jaemin Kim[2], Matthias Rüegg[1], Odysseas Chaliotis[1], Jon Märki[1], Nikolaos Bouklas[2], and Mahmut Selman Sakar[1*]

[1] Institute of Mechanical Engineering, Ecole Polytechnique Fédérale de Lausanne, 1015 Lausanne, Switzerland

[2] Sibley School of Mechanical and Aerospace Engineering, Cornell University, Ithaca, New York

* Corresponding author: selman.sakar@epfl.ch

Supplementary Figures

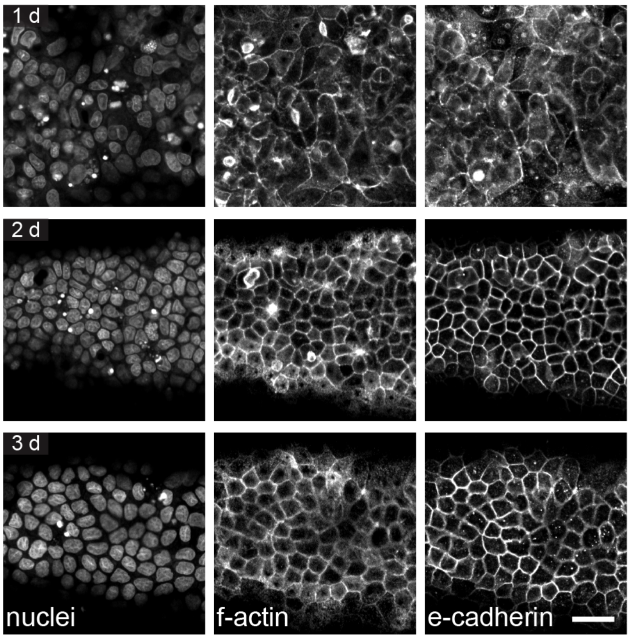

Figure S1. Epithelial cells form a coherent monolayer on the 3D collagen gel. Confocal micrographs showing the crowding and jamming of the epithelium over time (rows). Different columns show nuclei, F-actin, and E-cadherin staining (from left to right). Scale bar, 30 μm.

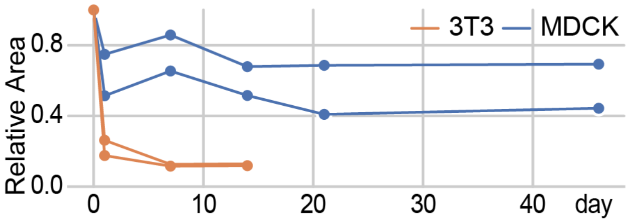

Figure S2. Representative graphs showing the projected area (xy plane) relative to the area of the microwell.

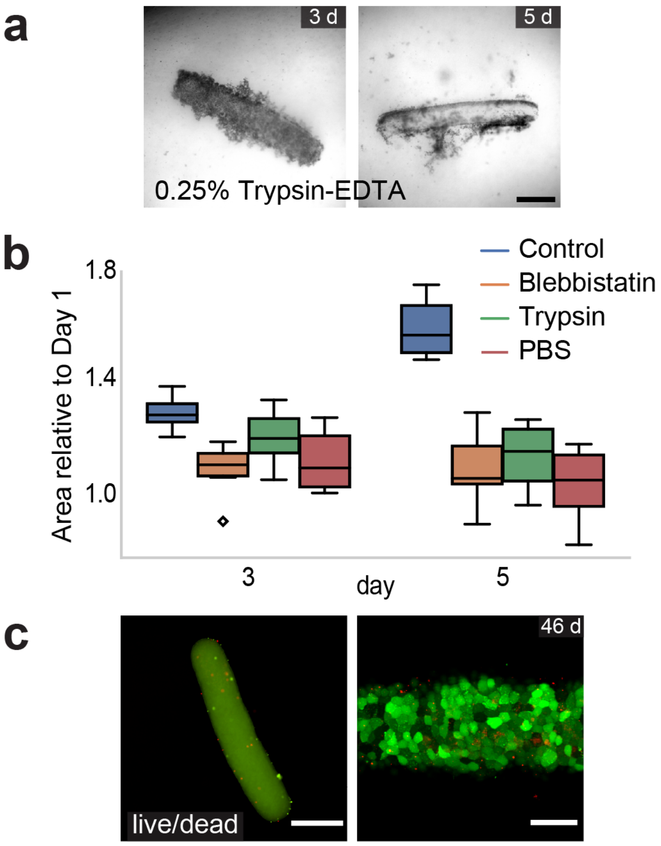

Figure S3. Tissue mechanics and morphology. (a) Representative images of a microtissue treated with 0.25% Trypsin-EDTA. Complete removal of the epithelium lead to a small increase in tissue size. Scale bar, 500 µm (b) Boxplot showing the vertical cross-sectional area of microtissues on days 3 and 5 normalized by the area at day 1. Tissues are treated with various pharmacological agents (N = 3 and n = 8). (c) Cell viability staining of microtissues (live, green and dead, red) 7 weeks after formation. Left: full-tissue view. Scale bar, 500 µm. Right: magnified view of a small area. Scale bar, 50 µm.

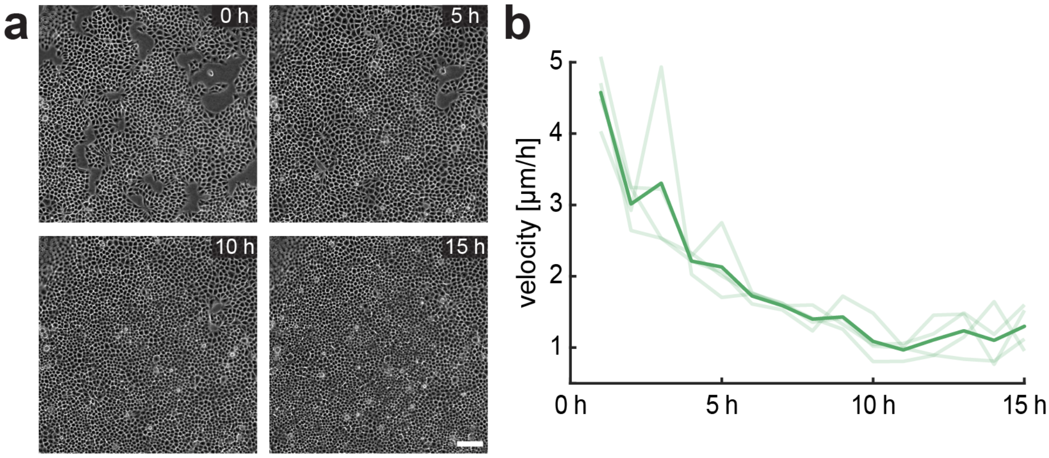

Figure S4. Jamming transition in an epithelial monolayer. (a) Representative micrographs of MDCK cells over time. The free areas are gradually filled with cells while they shrink in size. Scale bar, 100 µm. (b) Particle image velocimetry measurements quantify cell motion over time in an area of 850 x 850 µm. The graph shows the mean of all velocity values within a frame for every hour. Transparent lines show representative individual samples and the bold line represent the mean value.

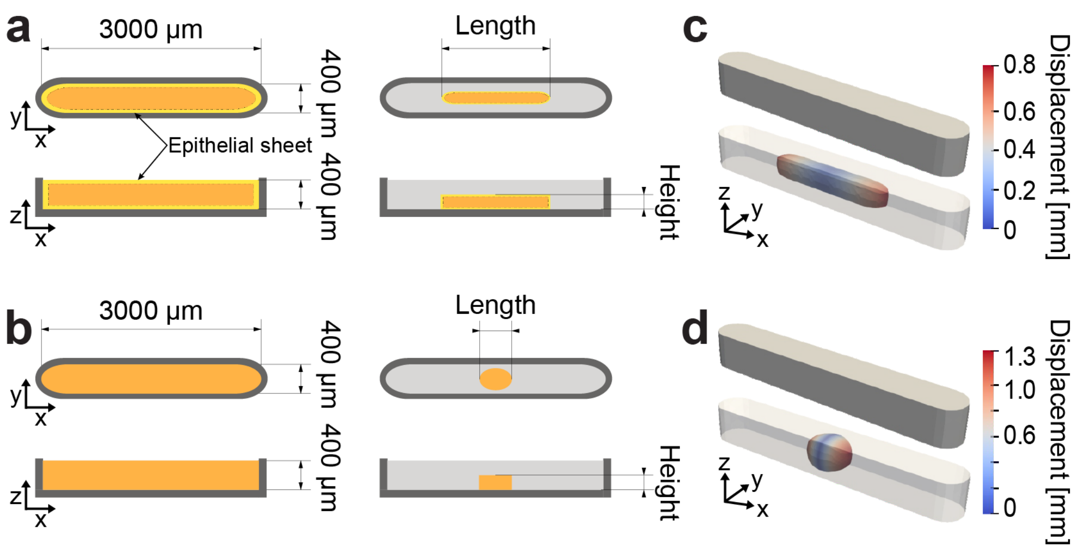

Figure S5. Finite element simulation of tissue shape. Schematic of microtissue formation that recapitulates gel coated with (a) epithelial cells and (b) fibroblasts. 3D view of the equilibrium shapes of the microtissues that are coated with (c) epithelial cells and (d) fibroblasts. At the initial state, the contractile moduli is set to zero. At the equilibrium state, the contractile moduli is set to their calibrated value. Color bar indicates displacement in mm.

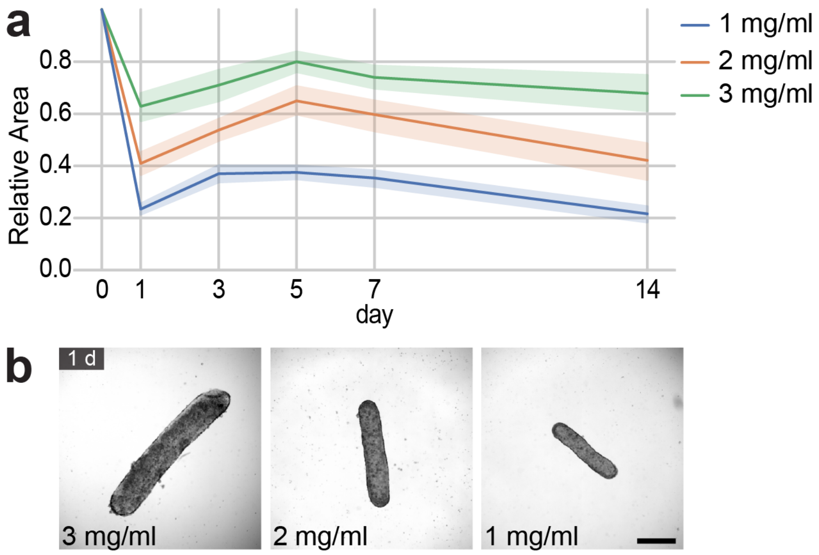

Figure S6. Tissue contraction and gel composition. (a) Line graph showing the vertical cross-sectional area of microtissues relative to the area of the microwell over days. Mean and 95% confidence intervals are shown for the measurements collected on different days (N = 3 and n = 12). (b) Representative images corresponding to different collagen concentrations. Scale bar, 500 µm

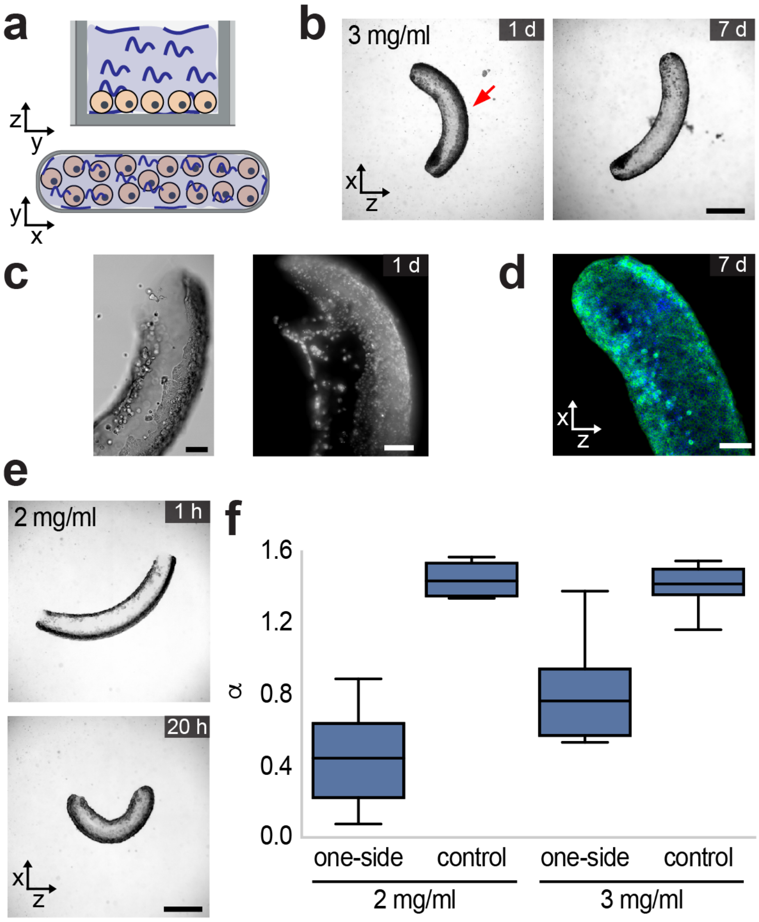

Figure S7. **Anisotropically engineered tissues show a higher curvature.** (a) Schematic illustration of tissue engineering. 2 million cells are seeded at the bottom of the well. (b) Representative images of microtissues engineered with 3 mg/ml collagen, 1 and 7 days after engineering. Red arrow indicates location of cells. (c) Within the first hours cells start to migrate towards the cell-free area. Brightfield image (left) and fluorescent image with dapi labeled nuclei (right) is shown. Scale bars, 100 µm. (d) Confocal micrograph showing f-actin (green) and nuclei (blue) of microtissue after 7 days. Scale bar, 100 µm. (e) Representative phase-contrast images showing tissues engineered with 2mg/ml collagen, 1 and 20 hours after engineering. Tissues bend towards the cell-free area due cell contraction. (f) Boxplot showing central angle α of tissues engineered anisotropically (one-side) or following the normal engineering protocol (control) (N = 1 and n = 12). Scale bars, 500 µm unless defined otherwise.

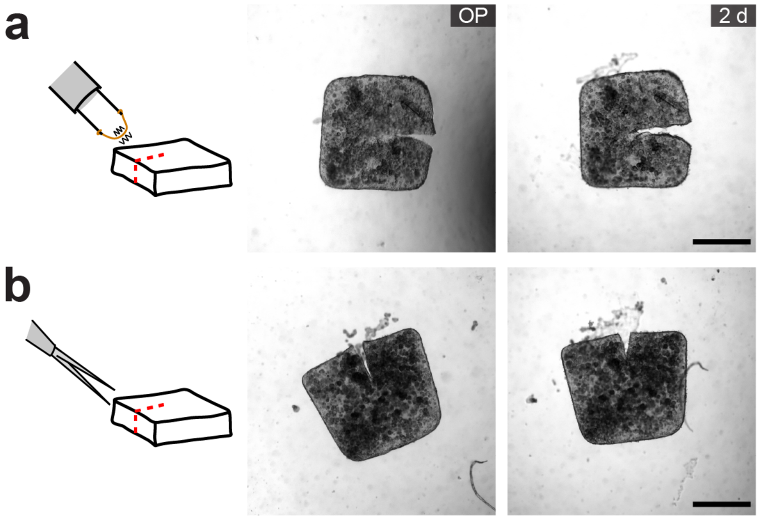

Figure S8. **Shape evolution in response to a full-thickness single cut.** The cut was performed either with (a) electro cautery device or (b) microscissors. Scale bars, 500 µm.

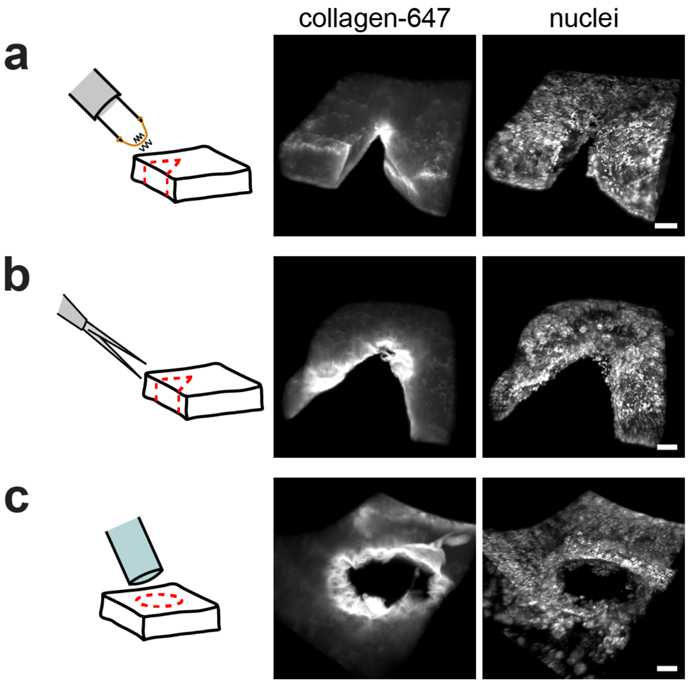

Figure S9. **The impact of surgical operations on the wound edge.** Schematics of the operations are shown on the left and the corresponding 3D reconstruction of light-sheet images are shown on the right. For the fluorescence imaging, the tissues were labelled for collagen (Center) and cell nuclei (Right). (a) The microtissue is cleanly cut with a microcautery device. High frequency vibration of a microwire cuts the biological material without leaving a detectable scar. (b) Cutting the microtissue with microscissors leads to plastic damage due to compressive stresses. (c) Piercing a hole into the centre of the tissue using a biopsy punch generates damage along the direction of the insertion. Scale bars, 100 µm.

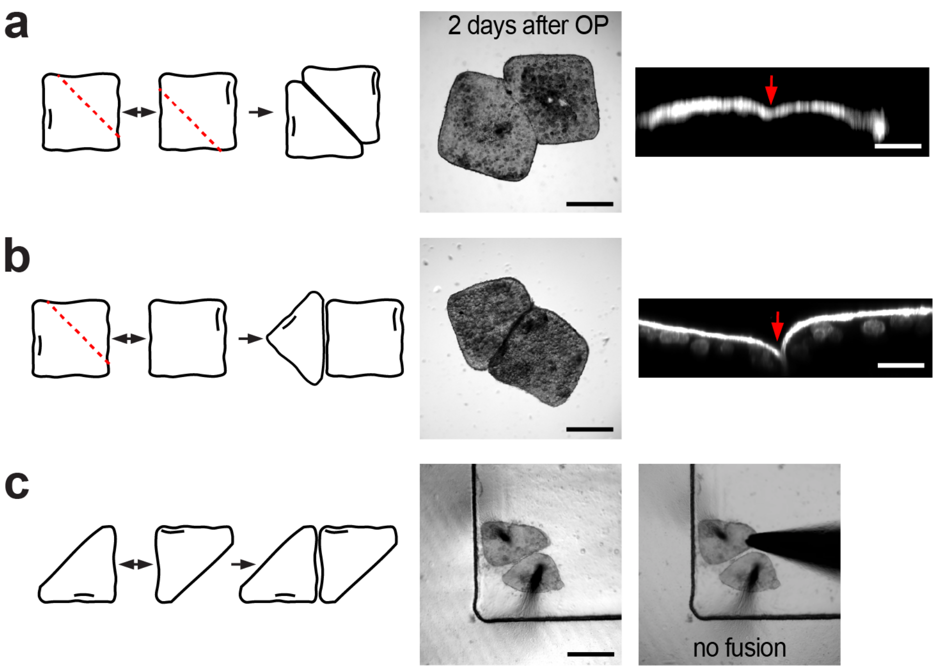

Figure S10. Proper tissue assembly is contingent on exposing the interfacing tissue parts. From left to right for (a) and (b): Schematic illustration of the operation, representative phase-contrast images of the assembled tissues (Scale bars, 500 µm), and f-actin staining showing the epithelium at the interface (Scale bars, 100 µm). Red arrows indicate the location where epithelial cells from two different pieces interface. (c) Two pieces of tissues are brought together without any surgical operation before the assembly. From left to right: Schematic illustration of the operation, representative phase-contrast image of the assembled tissues (Scale bar, 500 µm), and an image from the experiment where we physically detached the pieces from each other by gentle pushing them apart.

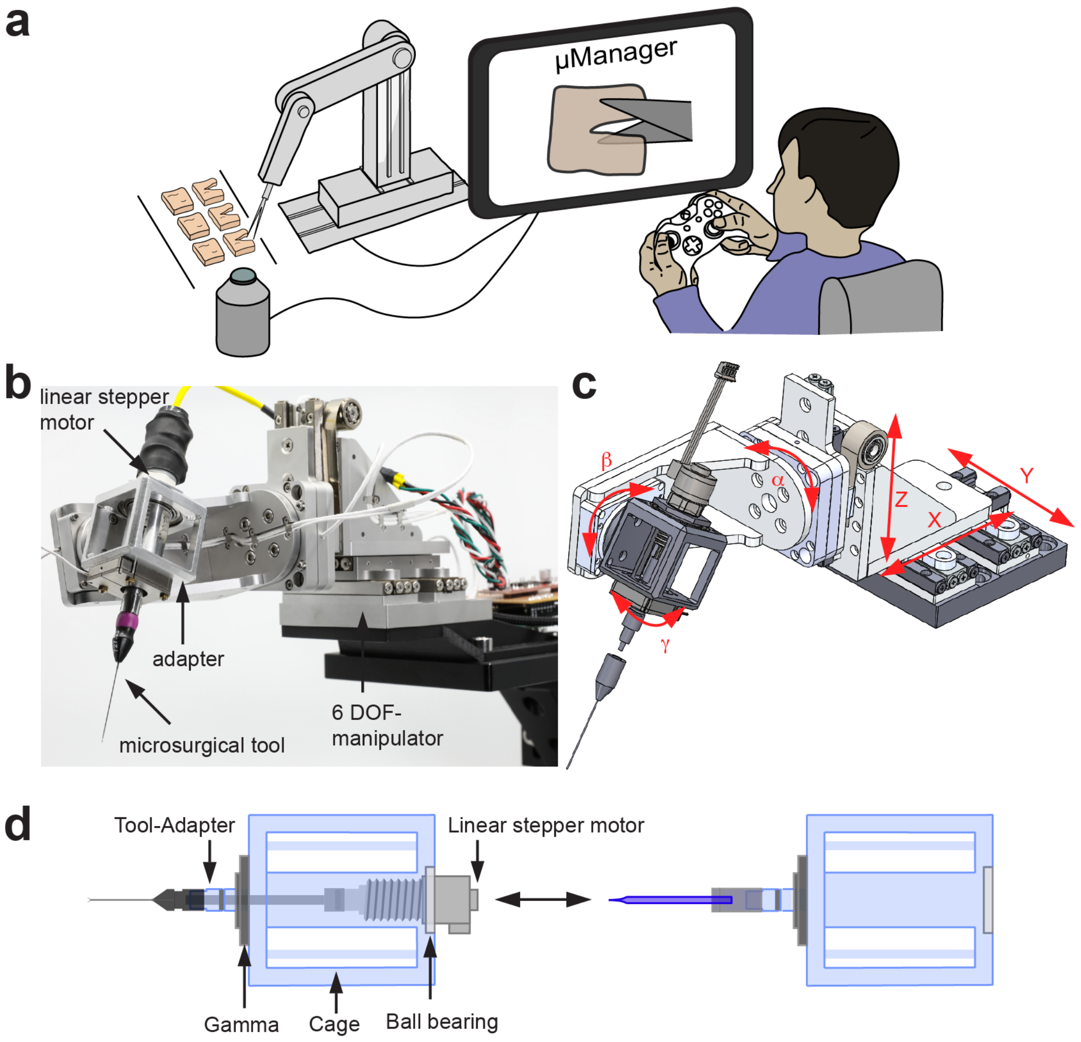

Figure S11. 6-DOF robotic micromanipulation system. (a) Schematic illustrating teleoperated manipulation on biological microtissues. The user controls the robotic manipulator with an Xbox controller, connected via the software interface µManager. The operation is done on a microscope with real-time feedback from a camera. (b) Photograph of the six-degree of freedom (6-DOF) system built from piezo-electric stick-slip actuators extended with a custom-made adapter for tool actuation. (c) CAD drawing of the robotic system with red arrows indicating the translational axes X, Y, Z and the rotational wrist composed of α, β and γ. (d) Section view of the adapter along with a disposable surgical instrument actuated by a linear stepper motor (Left), or along with a non-actuated tool (e.g. glass capillary) and without the stepper motor (Right).

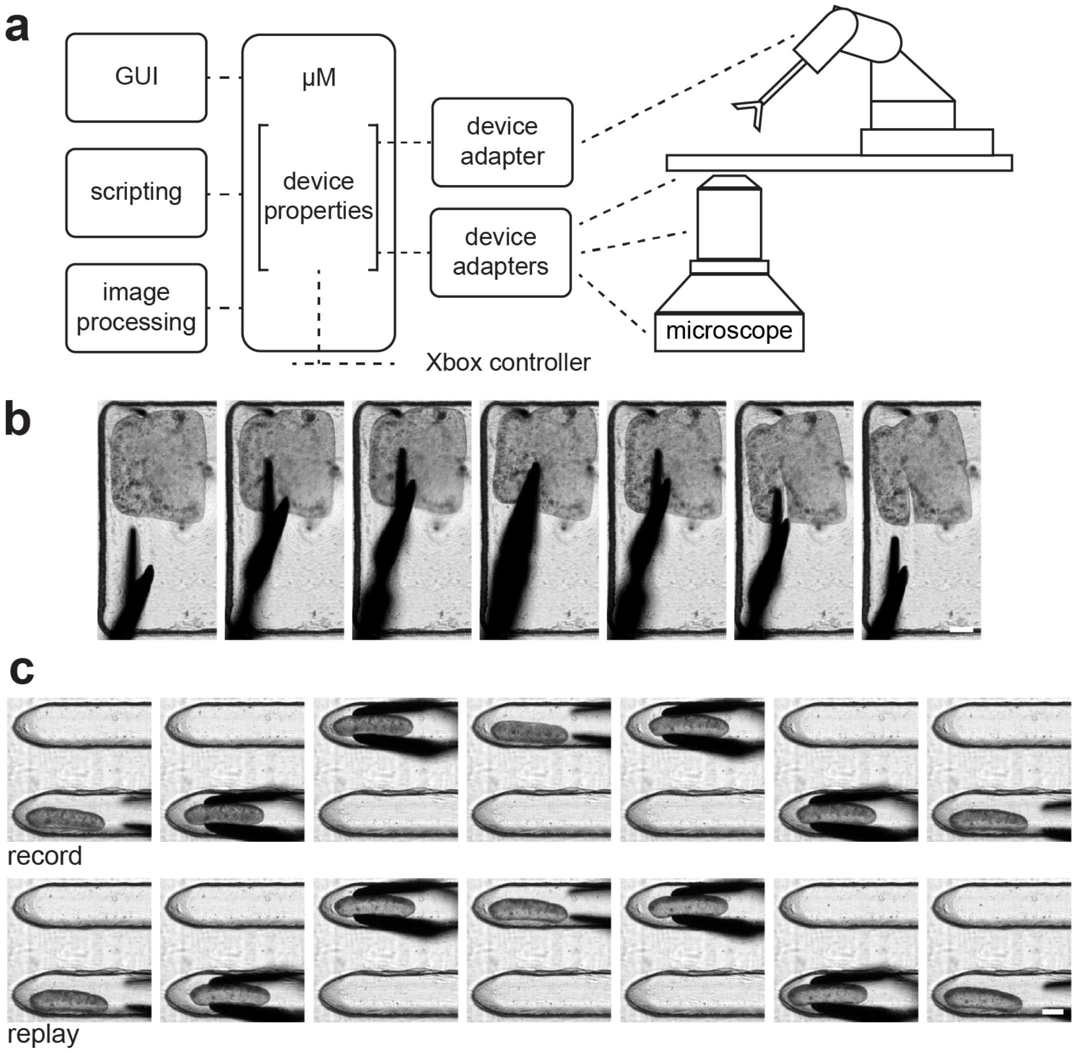

Figure S12. Robotic micromanipulation of microtissues. (a) Schematic illustration of the software architecture. The robot as well as other microscopy instruments communicate with the Core API from μManager via device adapters. Device adapters translate specific device driver API to common μManager plug-in interface. Each loaded device exposes a number of properties which can be read and changed via μManager. Teleoperation is performed using an Xbox controller connected to the μManager via a built-in plugin. μManager provides the graphical interface as well as scripting environment for control and real-time image processing. (b) Cutting a tissue using the robotic system equipped with microscissors. (c) Pick-and-place of tissues from one well to another using the robotic system equipped with tweezers. The operation was recorded and automatically repeated by the robot. Operations were recorded on an inverted microscope equipped with a 4X objective. Scale bars, 200 μm.

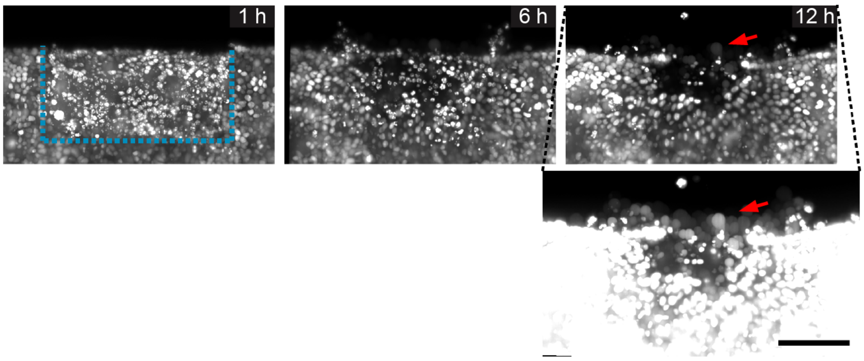

Figure S13. Cells around the illuminated area migrate towards the wound and extrude dead cells. The extrusion of dead cells from the tissue is monitored over time. Inset at 12 hours: Extruded dead cells have reduced expression of fluorescent nuclei label, and thus can be made visible by adapting brightness settings (red arrow). Scale bar, 50 µm

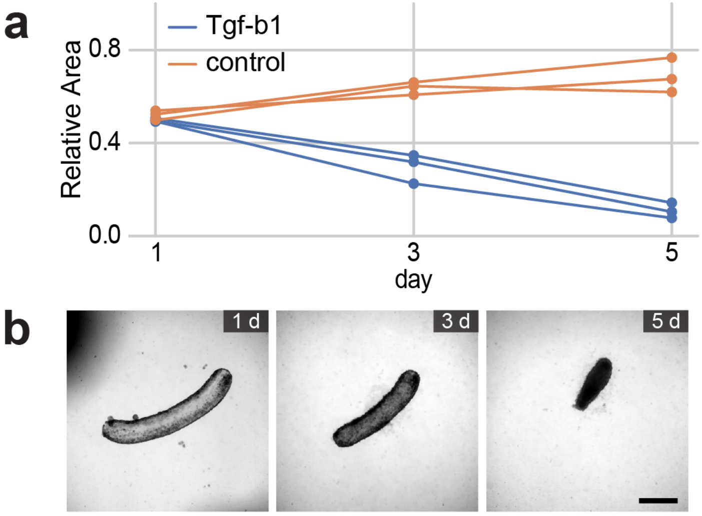

Figure S14. Treatment with growth factor leads to phase transition. (a) Linegraph showing the evolution of the projected area of tissues representing the control (orange) and treatment with Tgf- β1 (blue). The measured area is relative to the area of the initial well size. (b) Representative images of a microtissue treated with Tgf- β1 over time. Scale bar, 500 µm.

Supplementary Tables

Table S1. Parameters used in computer simulation to recapitulate shape of microtissues.

	E	ν	K	G	λ	η (kPa)
epithelial microtissue 3 mg/ml collagen	0.2	0.09	0.081	0.09	0.02	0.56
epithelial microtissue 2 mg/ml collagen	0.1	0.09	0.041	0.05	0.01	0.56
mesenchymal microtissue 3 mg/ml collagen	0.2	0.09	0.081	0.09	0.02	0.43

Table S2. Simulated and measured equilibrium sizes of tissues.

	Length	Width	Height
epithelial microtissue 3 mg/ml collagen			
Experiment	1890 +/- 263	324 +/- 30	-
Simulation	1884	298	297
epithelial microtissue 2 mg/ml collagen			
Experiment	1597 +/- 303	301 +/- 40	-
Simulation	1558	261	261
mesenchymal microtissue 3 mg/ml collagen			
Experiment	427 +/- 18	340 +/- 13	-
Simulation	430	349	347

Supplementary Note 1. A computational model of tissue morphogenesis driven by epithelial and mesenchymal cells residing on the tissue surface

We refer the reader to our previous work ([1,2]) for the general theory on the curvilinear kinematics of the surface and the volume of a 3D soft body. Specific considerations on the implementation of this work are introduced in the following formulations.

1. Kinematics

Let Ω_0 be a fixed reference configuration of a continuum body $\mathcal{B}$. We use the notation $\chi: \Omega_0 \to \mathbb{R}^3$ for the deformation of body $\mathcal{B}$. A motion χ is the vector field of the mapping $\boldsymbol{x} = \chi(\mathbf{X})$, of a material point in the reference configuration $\mathbf{X} \in \Omega_0$ to a position in the deformed configuration $\boldsymbol{x} \in \Omega$. The kinematics of a material point are described by

$$\mathbf{u}(\mathbf{X}, t) = \boldsymbol{x}(\mathbf{X}, t) - \mathbf{X} \tag{S1}$$

where $\mathbf{u}(\mathbf{X}, t)$ is the displacement vector field in the spatial description. The kinematics of an infinitesimal bulk element are described by

$$\mathbf{F}(\mathbf{X}, t) = \frac{\partial \chi(\mathbf{X}, t)}{\partial \mathbf{X}} = \nabla_{\mathbf{X}} \boldsymbol{x}(\mathbf{X}, t) \tag{S2}$$

$$\mathbf{F}^{-1}(\boldsymbol{x}, t) = \frac{\partial \chi^{-1}(\boldsymbol{x}, t)}{\partial \boldsymbol{x}} = \nabla_{\boldsymbol{x}} \mathbf{X}(\boldsymbol{x}, t) \tag{S3}$$

where $\mathbf{F}(\mathbf{X}, t)$ and $\mathbf{F}^{-1}(\boldsymbol{x}, t)$ are the deformation gradient and inverse deformation gradient, respectively. Note that $J(\mathbf{X}, t) = \mathrm{d}v/\mathrm{d}V = \det \mathbf{F}(\mathbf{X}, t)$ is the Jacobian determinant defining the ratio of a volume element between material and spatial configuration. Furthermore, we decompose the deformation gradient into a volumetric and an isochoric part ([3]).

$$\mathbf{F} = (J^{1/3}\mathbf{I})\bar{\mathbf{F}} \tag{S4}$$

where $J^{1/3}\mathbf{I}$ and $\bar{\mathbf{F}}$ represent the volumetric and isochoric parts of the deformation gradient $\mathbf{F}$. Note that we utilize $\{\bar{\bullet}\}$ to denote quantities associated with the isochoric part of the deformation gradient.

A motion of an arbitrary differential vector element can be mapped by the deformation gradient $\mathbf{F}$. However, a unit normal vector $\mathbf{N}$ in the reference configuration cannot be transformed into a unit normal vector $\mathbf{n}$ in the current configuration via the deformation gradient ([3]), motivating us to develop the

kinematics of an infinitesimal surface element ([4]). Note that we utilize $\{\widehat{\bullet}\}$ to denote the surface quantity bounded by outer surface denoted as $\partial\Omega_0$.

$$\hat{\mathbf{F}}(\mathbf{X},t) = \frac{\partial\chi(\mathbf{X},t)}{\partial\mathbf{X}}\cdot\hat{\mathbf{I}} = \hat{\nabla}_{\mathbf{X}}\boldsymbol{x}(\mathbf{X},t) \tag{S5}$$

$$\hat{\mathbf{F}}^{-1}(\boldsymbol{x},t) = \frac{\partial\chi^{-1}(\boldsymbol{x},t)}{\partial\boldsymbol{x}}\cdot\hat{\boldsymbol{\iota}} = \hat{\nabla}_{\boldsymbol{x}}\mathbf{X}(\boldsymbol{x},t) \tag{S6}$$

where $\hat{\mathbf{F}}(\mathbf{X},t)$ and $\hat{\mathbf{F}}^{-1}(\boldsymbol{x},t)$ are the deformation gradient and inverse deformation gradient, respectively. Note that $\hat{\mathbf{I}} = \mathbf{I} - \mathbf{N}\otimes\mathbf{N}$ and $\hat{\boldsymbol{\iota}} = \boldsymbol{i} - \mathbf{n}\otimes\mathbf{n}$ are the mixed surface unit tensors, where $\mathbf{I}$ and $\boldsymbol{i}$ are the unit tensors, and $\mathbf{N}$ and $\mathbf{n}$ are the outward unit normal vectors, in reference and current configuration, respectively. Note that $\hat{J}(\mathbf{X},t) = \mathrm{d}a/\mathrm{d}A = |\mathrm{cof}\,\mathbf{F}\cdot\mathbf{N}|$ is the Jacobian determinant defining the ratio of an area element between material and spatial configuration.

We introduce following bulk strain measures ([3]) as follows:

$$\mathbf{C} = \mathbf{F}^{\mathrm{T}}\mathbf{F} \quad \text{and} \quad I_1 = \mathrm{tr}(\mathbf{C}) \tag{S7}$$

$$\bar{\mathbf{C}} = \bar{\mathbf{F}}^{\mathrm{T}}\bar{\mathbf{F}} \quad \text{and} \quad \bar{I}_1 = \mathrm{tr}(\bar{\mathbf{C}}) \tag{S8}$$

where $\mathbf{C}$ and I_1 are the right Cauchy–Green tensor and the first principal invariant, and $\bar{\mathbf{C}}$ and $\bar{I}_1$ are the modified quantities for the isochoric deformation.

2. Equilibrium

The total potential energy functional $W(\chi)$ is defined as:

$$W(\chi) = \int_{\Omega_0}\Psi(\mathbf{F},\chi;\mathbf{X})\mathrm{d}V + \int_{\partial\Omega_0}\hat{\Psi}(\hat{\mathbf{F}},\chi;\mathbf{X})\mathrm{d}S - \int_{\Omega_0}\mathbf{B}\cdot\mathbf{u}(\chi;\mathbf{X})\mathrm{d}V - \int_{\partial\Omega_0}\mathbf{T}\cdot\mathbf{u}(\chi;\mathbf{X})\mathrm{d}S \tag{S9}$$

where $\mathbf{B}$ is the reference body force and $\mathbf{T}$ is the surface traction. An equilibrated configuration is obtained by minimizing this functional considering all admissible deformations $\delta\chi$. It is important to note that the strain energies $(\Psi,\hat{\Psi})$ can be varied depending on the epithelial and mesenchymal models so that the following sections can be written in a single formulation for brevity.

Following the derivation presented in [5], we can finally arrive at a set of localized force balance equations. Neglecting the inertial effect, the local form of linear and angular momentum balances for bulk and surface are defined by

$$\nabla_{\mathbf{X}} \cdot \mathbf{P} + \mathbf{B} = \mathbf{0} \quad \text{in } \Omega_0 \tag{S10}$$

$$\widehat{\nabla}_{\mathbf{X}} \cdot \widehat{\mathbf{P}} + \mathbf{T} - \mathbf{PN} = \mathbf{0} \quad \text{on } \partial\Omega_0 \tag{S11}$$

$$\mathbf{u} = \breve{\mathbf{u}} \quad \text{on } \partial\Omega_{0,\mathbf{u}} \tag{S12}$$

where $\breve{\mathbf{u}}$ is the prescribed displacement on the boundary $\partial\Omega_{0,\mathbf{u}}$. Note that a Neumann-type boundary condition is also defined on boundary curves, $[\![\widehat{\mathbf{P}}\widehat{\mathbf{N}}]\!] = 0$ where is the bi-normal vector to the boundary curve and $[\![\bullet]\!]$ indicates summation over surfaces intersecting on boundary curves ([4]).

3. Weak Form

For the finite element implementation, we need to obtain the weak form for our problem. By adding the constraint that the first variation of the total potential energy must be equal to zero $\delta W(\chi) = 0$, we obtain a weak form statement as

$$G = \int_{\Omega_0} \mathbf{P} : \nabla_{\mathbf{X}} \delta\mathbf{u} \, \mathrm{d}V + \int_{\partial\Omega_0} \widehat{\mathbf{P}} : \widehat{\nabla}_{\mathbf{X}} \delta\mathbf{u} \, \mathrm{d}S - \int_{\Omega_0} \mathbf{B} \cdot \delta\mathbf{u} \, \mathrm{d}V - \int_{\partial\Omega_0} \mathbf{T} \cdot \delta\mathbf{u} \, \mathrm{d}S = 0 \quad \square\, \delta\mathbf{u} \tag{S13}$$

where $\delta\mathbf{u}$ is the admissible deformation field.

We employed the open-source platform FEniCS ([6]), to implement the finite element simulation. We used the Scalable Nonlinear Equations Solvers (SNES) from the open-source toolkit PETSc ([7]), which provides numerical computations of a Newton-type iterative procedure to solve the nonlinear variational problem. Note that the values of η and γ should be ramped from zero to their prescribed values for numerical stability as the problem is highly nonlinear.

4. Constitutive Relations

To relate the active stresses with deformation, we must specialize our choice for the strain energy in the bulk and on the surface. We postulate that the bulk strain energy density can be decomposed into the passive and active parts as $\Psi = \Psi^p + \Psi^a$ and additionally the surface strain energy density $\widehat{\Psi}^a$. For the deformation of compressible tissue, we consider a passive bulk energy Ψ^p that captures the permanent elasticity of the collagen network, and for the contribution of cellular contractility, we can consider the

active bulk energy Ψ^a that accounts for contractile action of the epithelial sheet, and surface energy $\hat{\Psi}^a$ that accounts for the action of the mesenchymal cells on the surface of the ECM.

4.1. Passive ECM model

The passive strain energy density Ψ^p describes the elasticity of ECM. We choose the compressible hyperelastic model ([2,3]) for the ECM network of both epithelial and mesenchymal tissues.

$$\Psi^p = \frac{K}{2}(J-1)^2 + \frac{G}{2}(I_1 - 3 - 2\ln J) \tag{S14}$$

where K and G are the bulk and shear moduli.

4.2. Epithelial cell sheet

The action of the epithelial cells, which leads to the formation of a contractile elastic sheet encapsulating the ECM, is considered by the addition of an elastic surface layer of constant thickness. This elastic sheet has a bulk strain energy that is leading to contraction in a volumetric fashion within the layer. That is, the epithelial sheet has both a passive and an active strain energy contribution. The passive bulk energy Ψ_e^p introduces the elasticity of the sheet, and the active bulk energy Ψ_e^a leads to the contraction. We employ a slightly modified compressible hyperelastic model Ψ_e^p to account for the volumetric deformation of epithelial sheet different from the ECM network, and we postulate that the active bulk energy Ψ_e^a is a function of the change of the volume J.

$$\Psi_e^p = \frac{K}{2}(J-1)^2 + \frac{G}{2}(\bar{I}_1 - 3) \tag{S15}$$

$$\Psi_e^a = \eta J \tag{S16}$$

where η is a bulk contractile modulus (energy per unit volume) representing the contractility of epithelial cell sheet at the equilibrium state. Note that the subscript e denotes the strain energies acting in the epithelial cell sheet.

4.3. Fibroblasts residing on the gel surface

Following our previous work ([1,2]), we assume that the mesenchymal cell-ECM adhesion can be described through a surface strain energy generating constant surface stresses similar to fluid-like surface tension. Unlike the epithelial cell sheet, the mesenchymal cell-ECM adhesion does not form an elastic layer, but generates contractility on the free surface through an active surface energy $\hat{\Psi}^a$. We postulate that the surface energy $\hat{\Psi}^a$ is a function of the change of the surface area $\hat{J}$.

$$\hat{\Psi}^a = \gamma \hat{J} \tag{S17}$$

where γ is a surface contractile modulus (energy per unit area) representing the contractility of mesenchymal cells on the surface at the equilibrium state. No additional passive elasticity needs to be considered here as the mesenchymal cells have low cell-cell adhesion.

5. Finite Element Simulation

The reference (undeformed) state is defined when the contractile moduli are zero. Experimentally, this reference state corresponds to the initial state of the tissue right after the gelation of the collagen matrix and before the application of forces by encapsulated cells. The reference configuration for the finite element simulations represents the geometry shown in Figure S5a,b. The computational model is based on an equilibrium theory, and therefore it cannot capture the transient states of the morphogenetic process. The parameters were calibrated to the experimental measurements (length and height in Figure S5a,b) of a representative microtissue at equilibrium. For the epithelial cell simulation, the contractile bulk modulus is only applied in the epithelial sheet which has a uniform thickness ($t = 50\mu m$) at the initial state (Figure S5a). For the mesenchymal cell simulation, the entire surface is allowed to actively contract though a non-zero surface contractile modulus (Figure S5b). The center points are constrained in the finite element simulations of a free-floating microtissue so that the rigid body motion is prohibited. The final (deformed) state is defined when the bulk and surface contractile moduli η and γ reach their prescribed values, and no external loads are applied. Experimentally, this corresponds to the equilibrium state of the microtissues. The final configurations represent the equilibrium states in Figure S5c,d.

6. Parameter Calibration

The parameters of the two models are the bulk and shear moduli, K and G, and bulk and surface contractile moduli, η and γ, respectively. Collagen gels of concentration of 3mg/ml and 2 mg/ml have been reported to have a Young's modulus of $E = 0.2$ kPa and $E = 0.1$ kPa, respectively [8]. The bulk and shear moduli, K and G, are uniquely paired with a set of parameters for Young's modulus E and Poisson's ratio ν. Thus, starting from the experimental values for Young's modulus, E, we need to uniquely define Poisson's ratio, ν. We calibrated ν, η and γ through an error analysis between the simulation and experimental results, using the shape of the tissues formed with 3 mg/ml collagen. The calibrated values are: $\nu = 0.09$ ($K = 81$ Pa and $G = 92$ Pa), $\eta = 560$ Pa and $\gamma = 431$ mN/m. Table S1

has the values of all the parameters. Table S2 show the results from simulations and experiments regarding the equilibrated shape of the specimens.

Supplementary Note 2. Robotic micromanipulation system.

We present a multiple degrees of freedom (DOF) robotic manipulation system that provides the precision and dexterity required to operate on microscale biological samples (Figure S7). The system is designed to be modular to accommodate a variety of end-effectors commonly used in tissue manipulation. We addressed the heterogeneity challenge by implementing a "learn from the expert" paradigm. The system is capable of recording and repeating tasks performed during the teleoperation mode (Figure S8). The control software of the robot is developed within the micro-manager (µManager) framework, an open-source software that aims to bundle proprietary drivers of microscopy components using an application programming interface (API) [9]. µManager provides a graphical user interface (GUI) with plugins and libraries necessary to perform teleoperation and a scripting environment to program open-loop automated manipulation tasks. The overall framework is extendable to closed-loop control, particularly based on visual feedback thanks to the availability of a rich computer vision library.

1. Overall Concept

The actuators that are responsible for controlling the position and orientation of the end-effectors are expected to be compact and portable. Otherwise, the system would have compatibility issues with microscope stages and environmental chambers. Inspired by an ophthalmic microsurgery platform [11], we designed a 6-DOF robot with nested piezoelectric actuators that occupies a volume of 200 x 100 x 70 mm^3. We developed an adapter that allows mounting of actuated tools such as scissors and tweezers along with non-actuated tools such as dissection knifes, needles, and glass capillaries (Figure S7d).

For serial sample processing, the program has record and replay functions and is integrated into µManager. µManager provides a large ecosystem of existing device adapters for over 200 hardware drivers. The software platform specifies a device adapter API and unifies the driver interfaces from imaging devices to motorized stages of different vendors. The code is written in C++ programming language and it is amenable to future extensions such as solving the inverse kinematics problem and implementing elaborate referencing schemes. The device adapters are used as building blocks for various experimental units (e.g. microscope stage, syringe pump) on three different levels: (1) Individually compiled device adapters can be linked directly against a C/C++ application, independent from the µManager core functionality. (2) The GUI of µManager is used to design and script (beanshell)

experimental protocols including imaging and automated image processing. (3) Using one of the μManager wrappers for MATLAB or Python, the capabilities of specialized software can be tapped on such as machine learning (e.g. Pycro-Manager [10]).

2. Hardware

The robotic manipulation system is constructed from piezoelectric stick-slip actuators that have sub-micron positioning precision. It has three translation stages (X, Y, Z), two rotary actuators equipped with positional feedback (α (roll), β (pitch)) and a lightweight rotary stage without sensor (γ) in a 'T(X)-T(Y)-T(Z)-R(α)-R(β)-R(γ)'-configuration. To hold and actuate microsurgery tools, a custom designed adapter, mounted on β, incorporates the open-loop γ-stage for infinite axial rotation of the end-effector (Figure S7c).

End-effectors. The mechanism of actuating microsurgery tools such as microscissors and tweezers is based on the movement of a plunger against a passive spring-loaded mechanism. The movement of the plunger is coupled to the translation of a metal tube over the blades, controlling the opening and closing of the tool. We altered the original design and added a transmission to improve the accuracy and reduce play, a crucial upgrade for high-throughput automation. The adapter with the new design was micromachined from aluminium. An external cage houses a pivot-mounted tubular motor support that is rotated by the γ-stage. It contains a small lead screw stepper motor that acts as a piston to push the plunger into the mounted tool. Compared to the original design, overall complexity is reduced by stacking the end-effector directly to the γ-stage. The new design allows the use of a standard ball bearing with small diameter. The original design required a specialized ball bearing with large diameter and thin cross-section, which is significantly more expensive. The operation of the motor is controlled by a programmable single-board Arduino microcontroller. The motor can be quickly removed for the installation of non-actuated tools. We developed a specific tool adapter for glass capillaries connected to a microinjection system (PV830, World Precision Instruments). The adapter design allows the use of different capillary sizes with tubing connection.

Precision and accuracy. The translation stages are linear stages with a parallel-rail structure. One rail is the stick-slip actuator and the other a passive guide. Z has in addition a constant-force spring to compensate for the weight of the spherical wrist. α and β are closed-loop rotary stages and γ is an open-

loop rotary stage. The linear stages have a range of 40 mm and closed-loop resolution of 4 nm. They can be back-driven by applying a force of 5 N and apply maximum forces of around 4 N during motion. The rotary stages α and β have a resolution of 25 μ° (microdegree), can be back-driven by applying torques of, respectively, 15N-cm and 6 N-cm and apply maximum torque of, respectively, 6 N-cm and 3 N-cm. γ has a resolution of 3 m° (millidegree). The maximum stage speed is around 20 mm/s for the translational stages, 15 degree/s for α and β and around 45 degree/s for γ. The stepper motor has a per step increment of 0.02 mm delivering up to 35 N of force.

Referencing. The zero positions of the translation stages are set by their mechanical end stop. The α and β stages are equipped with a sensor that has nanometre resolution and a reference mark. While α has mechanical stops to prevent full revolution, safeguarding rotation in β required a specific software implementation. To ensure collision free referencing, the range is restricted to β = [0°, 90°] with a defined shutdown position at 0°. The open-loop rotary actuator γ does not have a mechanical end stop for operational reasons and is currently excluded from the referencing operation.

3. Software

The Smaract MCS controller unit handles the host communication and controls all stages. A μManager hub object is used as a single communication port to the controller to implement all cross-channel logic (Figure S8a). The linear stage class of μManager is used for all six stages due to the lack of a rotary stage class. Although the Arduino microcontroller has a simple serial connection protocol that is used by a generic device adapter class, it is accessed by the hub object to retrieve stepper motor positions. Device adapters communicate with μManager by exposing a number of properties which μManager can, respectively, read or write and potentially trigger an action of the connected device. A property is a field consisting of a name and a value. Properties can be accessed within the built-in GUI, via the beanshell scripting environment, or implemented plugins. We developed a user-friendly μManager-based GUI Plugin for manual command entry and retrieval of recorded data.

Motion control. Teleoperation is performed using an Xbox controller connected to the μManager with the ASI Gamepad plugin (https://micro-manager.org/ASI_Gamepad_Plugin). Xbox controllers are optimised for multiple DOF motion control. Button assignment is customizable, depending on the specifics of the manipulation task. There are two joysticks and we assigned them to control either the

translational stages or the spherical wrists. Upon displacement of the joystick, a discrete property value between -1 (left or down) and 1 (right or up) is sent to the corresponding stage. With increasing joystick displacement, the corresponding stage moves at around 25% of the maximum speed and linearly increases to 100%. To eliminate the errors accidentally caused by hand shaking, a displacement threshold is set. This way, parasitic movements occurring with less than a given relative movement amplitude are filtered out.

For tool actuation, the stepper motor is controlled using either a dynamic or a discrete mode. Dynamic control follows the same principle as stage movement, whereas an intensity vector that linearly goes from the zero to the maximum position is assigned to a trigger on the Xbox controller. Upon trigger release the stepper motor goes back to the zero position. The discrete method divides the range into steps and a trigger of a button changes a property value that indicates the discrete position of the stepper motor. The zero position, step range, and maximum position of the stepper motor are set and can be changed within the software.

Speed layer. Depending on the type of experiment and location of the manipulator, a variety of velocities or sensitivities are required, respectively. Speed layer functionality was implemented as a property value (1, 10, 100, or 1000), which acts as a simple multiplication factor of the speed variable that is computed on each movement based on the joystick displacement.

Motion recording and replay. The manipulator is equipped with a teach-in and replay functionality. Recording mode is activated by changing a Boolean property value. On activation, each change of position triggers the printing of all seven positions into a text file. The replay function runs the motion pattern from a selected text file. The motion pattern can be replayed from 0.1 up to 10 times of the original speed and offsets can be added to the translation stages.

Supplementary References

[1] KIM, Jaemin, et al. A model for 3D deformation and reconstruction of contractile microtissues. Soft Matter, 2021.

[2] MAILAND, Erik, et al. Surface and bulk stresses drive morphological changes in fibrous microtissues. Biophysical journal, 2019, 117.5: 975-986.

[3] HOLZAPFEL, A. Gerhard. Nonlinear solid mechanics II. 2000.

[4] STEINMANN, Paul. On boundary potential energies in deformational and configurational mechanics. Journal of the Mechanics and Physics of Solids, 2008, 56.3: 772-800.

[5] JAVILI, A.; STEINMANN, Paul. A finite element framework for continua with boundary energies. Part II: The three-dimensional case. Computer Methods in Applied Mechanics and Engineering, 2010, 199.9-12: 755-765.

[6] LOGG, Anders; MARDAL, Kent-Andre; WELLS, Garth (ed.). Automated solution of differential equations by the finite element method: The FEniCS book. Springer Science & Business Media, 2012.

[7] BALAY, Satish, et al. PETSc users manual. 2019.

[8] AREVALO, Richard C., et al. Size-dependent rheology of type-I collagen networks. Biophysical journal, 2010, 99.8: L65-L67.

[9] EDELSTEIN, Arthur, et al. Computer control of microscopes using µManager. Current Protocols in Molecular Biology, 2010, Suppl. 92:14.20.1-14.20.17.

[10] PINKARD, Henry, et al. Pycro-Manager: open-source software for customized and reproducible microscope control. Nature Methods, 2021, 18, 226-228.

[11] NAMBI, Manikantan, et al. A Compact Telemanipulated Retinal-Surgery System that Uses Commercially Available Instruments with a Quick-Change Adapter. Journal of Medical Robotics Research, 2016, 01, 1630001.

Supplementary Movies

Movie S1. Time-lapse movie showing cell motion on 2D collagen substrate over 15 hours**.** Epithelial cells, but not mesenchymal cells, undergo dynamic arrest.

Movie S2. Time-lapse movie recorded by a light-sheet microscope showing the collective migration of epithelial cells into the free area. Cell nuclei is labelled blue and f-actin is labelled green.

Movie S3. In the first part of the movie, two consecutive cuts are performed on a tissue using the robotic system equipped with microscissors. In the second part, a capsule-shaped tissue is carried from one well to another using the robotic system equipped with tweezers. The first operation performed by the user is recorded (indicated by 'record') and the remaining runs were replayed by the computer.

Movie S4. Time-lapse movie recorded by a light-sheet microscope showing epithelial cells that move into the illuminated area and extrude dead cells.

Movie S5. Time-lapse movie recorded by a light-sheet microscope showing the folding of the tissue initiated by a phototoxic molecule and structure plane illumination. Formation of creases are visible on both sides of the illuminated area as a manifestation of local collagen compaction.
